# Site-specific incorporation of a fluorescent nucleobase analog enhances i-motif stability and allows monitoring of i-motif folding inside cells

**DOI:** 10.1093/nar/gkae106

**Published:** 2024-02-16

**Authors:** Bartomeu Mir, Israel Serrano-Chacón, Pedro Medina, Veronica Macaluso, Montserrat Terrazas, Albert Gandioso, Miguel Garavís, Modesto Orozco, Núria Escaja, Carlos González

**Affiliations:** Instituto de Química Física ‘Blas Cabrera’. CSIC. Serrano 119. 28006 Madrid. Spain; Inorganic and Organic Chemistry Department. Organic Chemistry Section and IBUB. University of Barcelona, Martí i Franquès 1-11, 08028 Barcelona. Spain; Instituto de Química Física ‘Blas Cabrera’. CSIC. Serrano 119. 28006 Madrid. Spain; Institute for Research in Biomedicine (IRB Barcelona). The Barcelona Institute of Science and Technology (BIST). 08028 Barcelona. Spain; Institute for Research in Biomedicine (IRB Barcelona). The Barcelona Institute of Science and Technology (BIST). 08028 Barcelona. Spain; Departament de Bioquímica i Biomedicina. Facultat de Biologia. Universitat de Barcelona. 08028 Barcelona. Spain; Institute for Research in Biomedicine (IRB Barcelona). The Barcelona Institute of Science and Technology (BIST). 08028 Barcelona. Spain; Institute for Research in Biomedicine (IRB Barcelona). The Barcelona Institute of Science and Technology (BIST). 08028 Barcelona. Spain; Inorganic and Organic Chemistry Department. Organic Chemistry Section and IBUB. University of Barcelona, Martí i Franquès 1-11, 08028 Barcelona. Spain; Institute for Research in Biomedicine (IRB Barcelona). The Barcelona Institute of Science and Technology (BIST). 08028 Barcelona. Spain; Instituto de Química Física ‘Blas Cabrera’. CSIC. Serrano 119. 28006 Madrid. Spain; Institute for Research in Biomedicine (IRB Barcelona). The Barcelona Institute of Science and Technology (BIST). 08028 Barcelona. Spain; Departament de Bioquímica i Biomedicina. Facultat de Biologia. Universitat de Barcelona. 08028 Barcelona. Spain; Inorganic and Organic Chemistry Department. Organic Chemistry Section and IBUB. University of Barcelona, Martí i Franquès 1-11, 08028 Barcelona. Spain; Instituto de Química Física ‘Blas Cabrera’. CSIC. Serrano 119. 28006 Madrid. Spain

## Abstract

The i-motif is an intriguing non-canonical DNA structure, whose role in the cell is still controversial. Development of methods to study i-motif formation under physiological conditions in living cells is necessary to study its potential biological functions. The cytosine analog 1,3-diaza-2-oxophenoxazine (tC^O^) is a fluorescent nucleobase able to form either hemiprotonated base pairs with cytosine residues, or neutral base pairs with guanines. We show here that when tC^O^ is incorporated in the proximity of a G:C:G:C minor groove tetrad, it induces a strong thermal and pH stabilization, resulting in i-motifs with *T*_m_ of 39ºC at neutral pH. The structural determination by NMR methods reveals that the enhanced stability is due to a large stacking interaction between the guanines of the tetrad with the tC^O^ nucleobase, which forms a tC^O^:C^+^ in the folded structure at unusually-high pHs, leading to an increased quenching in its fluorescence at neutral conditions. This quenching is much lower when tC^O^ is base-paired to guanines and totally disappears when the oligonucleotide is unfolded. By taking profit of this property, we have been able to monitor i-motif folding in cells.

## Graphical Abstract

**Graphical Abstract:**
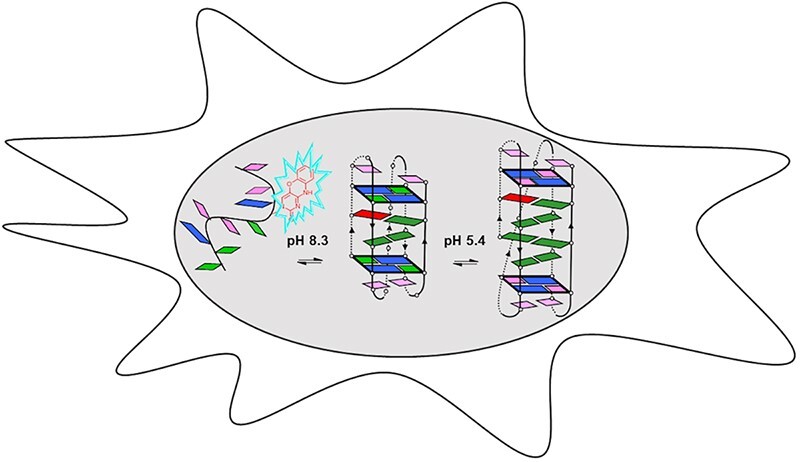

## Introduction

The i-motif (1) (or i-DNA) is a non-canonical DNA structure that is attracting interest due to its applications in nanotechnology and its possible implication in biological processes (2–4). The i-motif is a four-stranded structure comprising two parallel-stranded DNA duplexes that are intercalated in an anti-parallel orientation and stabilized by hemiprotonated C:C^+^ base pairs. Although i-motif structures are more stable at acidic conditions, there are an increasing number of sequences able to fold into stable i-motifs at neutral pH (5–10). Additionally, bioinformatic searches have revealed that some i-motif forming sequences are prevalent in the human genome (8,11), and numerous *in vitro* studies have found i-motifs in DNA sequences that are involved in biological processes like gene transcription (12–14), DNA synthesis (15), telomere (16) and centromere (17) maintenance, among others. All these findings, together with the recent observation of i-motifs in human cells (18,19) have boosted the interest in these structures. However, despite significant progress made in this field, further research is required to fully understand the roles of i-motifs in living cells. High-resolution microscopy techniques based on fluorescence spectroscopy are essential for these studies.

**Figure 1.**
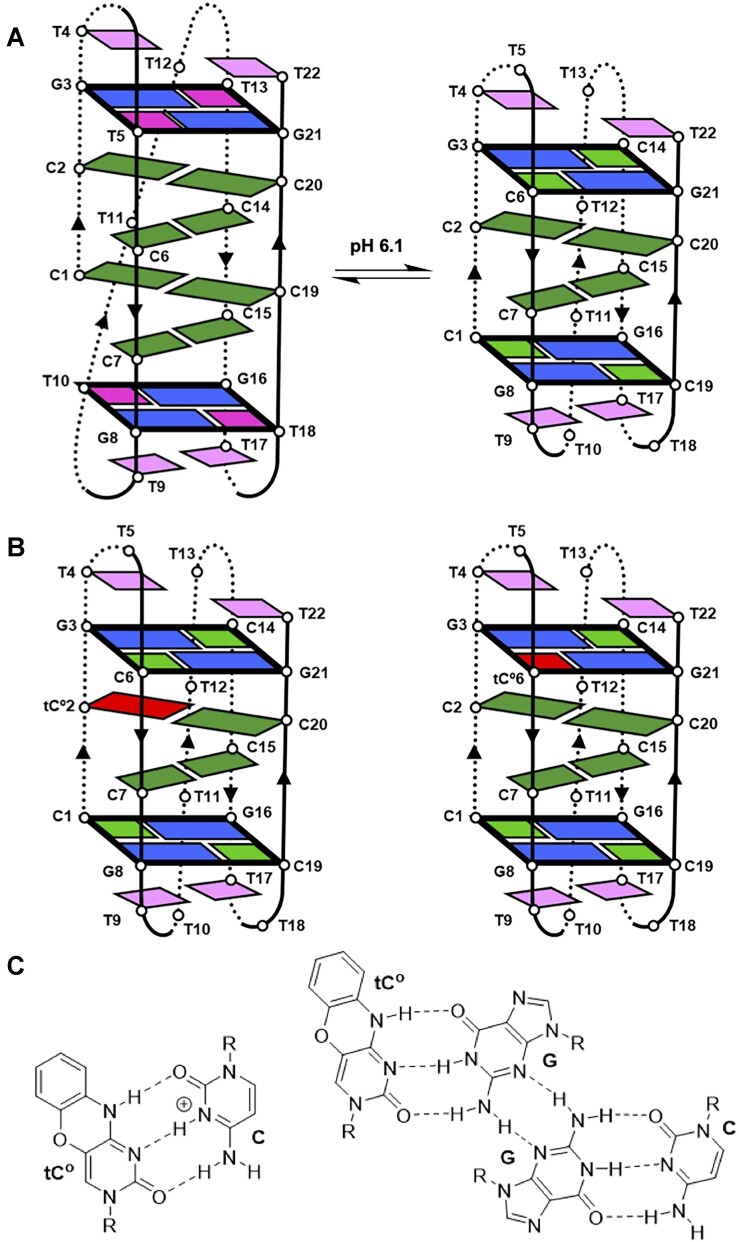
(**A**) Schematic representation of the pH-dependent species of NN4. (**B**) Expected structures adopted at neutral pH by the sequences NN4_tC^O^2 (left) and NN4_tC^O^6 (right). (**C**) tC^O^:C^+^ base pair and WC tC^O^:G base pair in a G:C:G:tC^O^ minor groove tetrad.

Fluorescent nucleobases have emerged as an extremely useful tool for nucleic acid detection and for studying nucleic acid interactions, folding, and dynamics (20). Of particular interest are those analogs that, while retaining their ability to form canonical base-pairs in duplexes, are also able to form other non-canonical structures. The cytosine analog 1,3-diaza-2-oxophenoxazine (tC^O^; Figure 1) belongs to this family of nucleobase analogs (21), as it is able to base pair with guanines forming Watson–Crick hydrogen bonds (22), and also with cytosines through the formation of hemiprotonated base pairs (23,24). tC^O^ and related cytidine analogs (tC, i-clamp (25) and DMAC (26)) have been used to study i-motif folding and to monitor i-motif/duplex transition kinetics. pH sensors based on these i-motif transitions have been proposed (23,25–27). Among all these cytosine analogs, tC^O^ exhibits the best quantum yield with absorption maximum at 370 nm (29). pH, salt, and temperature have very limited effects on their photophysical properties (29), and it has been reported that tC^O^ or related analogs do not affect the secondary structure of B-DNA upon hybridization (30,31). The high brightness displayed by tC^O^ upon formation of G:tC^O^ WC-like base pairs affords remarkably low quenching of the fluorescence signal in a B-DNA duplex environment (31). However, when incorporated into an i-motif-forming sequence, the formation of tC^O^:C^+^ base pairs causes a substantial quenching of the fluorescence signal (23,24). tC° can also be strategically used in FRET systems as donor combined with the FRET-acceptor analogue tC^nitro^ (32). In general, tC^O^ has a neutral or destabilizing effect on i-motif stability (23,24), and slightly stabilizing when incorporated in B-form DNA helices (30). Very recently, tC^O^ has been also incorporated in RNA molecules and used to monitor RNA processing in cells (33).

In this paper, we investigate the use of tC^O^ as a local probe for monitoring the folding and unfolding processes of i-motifs, as well as the conformational changes associated with the interconversion equilibrium between different i-motif structures. Our focus is on the recently reported structure of **NN4** (10), which sequence, d(CCGTTCCGT-TTTT-CCGTTCCGT), belongs to a unique set of repetitive sequences prevalent in regulatory regions of the human genome (8). Despite their low number of cytosines, these sequences fold into stable i-motifs under physiological conditions, thanks to the formation of two minor groove tetrads (MGTs) at the two ends of the i-motif. MGTs are the result of the association of two base pairs through their minor groove side, and have been observed with different arrangements of Watson–Crick base-pairs (34–36) or G:T mismatches (37–39). These tetrads are non-planar and, consequently, incompatible with G-tetrads (40). However, they have been identified in other non-canonical four-stranded DNA structures resulting from the self-association of two short oligonucleotides (41,42). In all these structures, MGTs are connected by short loops comprising one to three residues (43). The minor groove orientation between the two base pairs results in a very close proximity between phosphate groups in the backbones of different strands. This is a common feature shared with i-motifs, and, in fact, MGTs have been observed in several i-motif structures. MGTs are compatible with i-motifs, and recent studies have shown that they are excellent capping elements, inducing strong pH and thermal stabilization in i-motifs (8,10). This stabilization is due to the interaction between the tetrad and the adjacent positively charged C:C^+^ base pair, which results in a dramatic shift in the effective p*K*_a_ of these cytosines (10). The case of **NN4** is particularly interesting since it folds into two different i-motif species depending on the pH. At neutral pH, **NN4** folds into an i-motif structure with two C:C^+^ base pairs stabilized by two capping G:C:G:C minor groove tetrads, whereas at acidic pH, it adopts an alternative i-motif structure with four C:C^+^ base pairs and two G:T:G:T minor groove tetrads (Figure 1A). The transition between the two structures is driven by the protonation state of the cytosines involved in the G:C:G:C tetrad (10).

The simultaneous presence of cytosines in different protonation states in the same sequence makes **NN4** an excellent system for exploring the effects of tC^O^ incorporation in different chemical environments. This can be achieved by substituting either hemiprotonated cytosines, involved in C:C^+^ base pairs, or neutral ones, involved in G:C base pairs. Therefore, in this study, we investigate the structure and pH-dependent fluorescence properties of the oligonucleotides **NN4_tC^O^2** and **NN4_tC^O^6** (See Table 1 for the sequences) which incorporate tC^O^ in a cytosine position involved in C:C^+^ base pair or in a G:C:G:C minor groove tetrad, respectively (Figure 1B, C).

**Table 1.** Oligonucleotide sequences and *T*_m_ values for NN4, NN4_tC^O^2 and NN4_tC^O^6 at different pH. 25 mM phosphate buffer, [oligonucleotide]= 2 μM. Estimated error ± 0.5 (ºC)

Name	Sequence	*T*_m_ (ºC) pH 5	*T*_m_ (ºC) pH 7
**NN4**	d(CCGTTCCGT-TTTT-CCGTTCCGT)	53.3	29.2
**NN4_tC^O^2**	d(C**tC^O^**GTTCCGT-TTTT-CCGTTCCGT)	57.7	38.8
**NN4_tC^O^6**	d(CCGTT**tC^O^**CGT-TTTT-CCGTTCCGT)	53.4	30.7

## Materials and methods

### Oligonucleotides synthesis

Oligodeoxynucleotides **NN4_tC^O^2** and **NN4_tC^O^6** were synthesized on an ABI 3400 DNA synthesizer by using standard solid-phase phosphoramidite chemistry at 1 μmol scale. BTT 0.3 M in anhydrous ACN was used as activator agent and the coupling time for the tC^O^ residue was enlarged compared to the other nucleobases (900 s). Cleavage from the solid support and nucleobases deprotection were carried out with concentrated aqueous ammonium hydroxide at 55ºC for 12 h. Crude DMT-off products were purified by ion-exchange HPLC (NucleoPac PA-100 column from Dionex, 250 × 4 mm, 13 μm-diameter). Purification conditions: eluent A = 1 M NaCl 10% ACN, eluent B = 10% ACN, 25–40% A in 30 min, 1.5 ml/min. Purified oligonucleotides where afterwards analyzed by reverse-phase HPLC obtaining final products 94–97% pure. Medium-to-low final yields were obtained (12–16%) by UV quantification. Oligonucleotides were further desalted using Amicon® Ultra centrifugal devices.

### Mass spectrometry

MS-MALDI-TOF spectra of **NN4_tC^O^2** and **NN4_tC^O^6** were acquired in the negative ion mode on an ABSciex 4800 plus device (see Supplementary Figure S27). Samples were prepared by mixing 1μl of oligonucleotide solution (100–500 μM) with 1 μl of ammonium citrate (50 mg/ml) and allowed to interact for few seconds. Next 1 μl of the mixture and 1 μl of the matrix (2,4,6-trihidroxyacetophenone, THAP, 10 mg/ml in H2O/ACN 1:1) were mixed and deposited onto the plate.

### CD, UV and fluorescence spectroscopy

Circular dichroism spectra were recorded on a Jasco J-815 device coupled to a Peltier Jasco CDF-4265 accessory. CD spectra were recorded at different temperatures or pH values, scanning from 320 to 220 nm. Each spectrum is the result of three accumulations. Before starting the acquisition, the samples were allowed to stabilize for 5 min inside the instrument. The blank correction was done, after checking that the buffer does not exhibit any ellipticity, by deducting a straight baseline corresponding to the ellipticity at 320 nm.

UV spectra were recorded on a Jasco V-730 spectrophotometer fitted with a thermostated cell holder. For UV melting curves, renatured samples stored at 4ºC were allowed to stabilize at the starting temperature for 10 min. The variation of the absorbance was monitored at a fixed wavelength at a 0.5ºC/min temperature scan rate.

Fluorescence spectra were recorded on a Photon Technologies International spectrofluorometer equipped with a xenon lamp and a four-position carrousel thermoregulated by an external bath. Fluorescence spectra at different temperatures were recorded allowing the sample to stabilize for 5 min before starting each acquisition. Excitation spectra were recorded from 230 to 420 nm, detecting at 450 nm. The emission spectra were recorded from 380 to 580 nm, prior excitation at 370 nm.

Samples were suspended in 25 mM sodium phosphate buffer and adjusted to desired pH with concentrated HCl or NaOH aliquots. Samples were initially heated at 90°C for 5 min, and slowly allowed to cool to room temperature and stored at 4°C until use. For pH titration experiments (CD and fluorescence), the pH was adjusted by adding aliquots of concentrated solutions of HCl or NaOH. Molar extinction coefficients of the tC^O^-containing sequences (Supplementary Table S6) were calculated as reported by Sandin et al (31). pH data points were plotted and fitted with Boltzmann sigmoidal fits using Origin software.

### NMR

Samples for NMR experiments were dissolved in 9:1 H_2_O/D_2_O (25 mM sodium phosphate buffer). Experiments were carried out at different pH values, ranging from 4 to 7. The pH was adjusted by adding aliquots of concentrated solution of either DCl or NaOD. All NMR spectra were acquired on Bruker spectrometers operating at 600 and 800 MHz, equipped with cryoprobes and processed with the TOPSPIN software. NOESY spectra in 9:1 H_2_O/D_2_O were acquired with mixing times of 150 and 250 ms. TOCSY spectra were recorded with the standard MLEV-17 spin-lock sequence and a mixing time of 80 ms. The spectral analysis program SPARKY (44) was used for semiautomatic assignment of the NOESY cross-peaks and quantitative evaluation of the NOE intensities.

### NMR constraints and structural calculations

Qualitative distance constraints were obtained from NOE intensities. NOEs were classified as strong, medium or weak, and distances constraints were set accordingly to 3, 4 or 5 Å. In addition to these experimentally derived constraints, hydrogen bond and planarity constrains for the base pairs were used. Due to the relatively broad line-widths of the sugar proton signals, J-coupling constants were not accurately measured, but only, roughly estimated from DQF-COSY cross-peaks. Loose values were set for the sugar dihedral angles δ, ν_1_ and ν_2_ to constrain deoxyribose conformation to North or South domain as described in previous studies on related molecules (10,45).

Partial atomic charges for tC^O^ were calculated using the RESP model (46) after geometry optimization. The electrostatic potential energy were carry out at the Hartree–Fock level of theory using the 6–31G(d) basis set for consistency with other atomic charges in the AMBER force field (47). The sugar was replaced with a methyl group, which Cartesian coordinates were restrained at 0.0631 eu. This made the net charge of the base the same as the cytosine one in the AMBER force field. New force field parameters necessary were obtained from BSC1 (48) or GAFF (49). The parameters are available on the AMBER parameters database of the University of Manchester (amber.manchester.ac.uk).

Structures were calculated with the program CYANA 3.0 (50) and further refined with the SANDER module of the molecular dynamics package AMBER 18.0 (51). Resulting CYANA structures were taken as starting points for the AMBER refinement, consisting of a heating simulation, followed by plain simulations of 500 ps each. Long-range electrostatic interactions were evaluated with the Particle Mesh Ewald method. The BSC1 force field (48) was used to describe the DNA, and the TIP3P model (52) to simulate water molecules. Analysis of the representative structures was carried out with the program MOLMOL (53) and X3DNA (54). Coordinates are deposited in the PDB data bank (code 80FC).

### Fluorescence spectroscopy

For fluorescence spectroscopy experiments, HeLa cells (cultured in Dulbecco's Modified Eagle Medium supplemented with 10% fetal bovine serum) were seeded at a density of 50.000 cells per well on 24-well plates (Nunclon, Thermo Fisher Scientific) and incubated at 37ºC in a humidified atmosphere with 5% CO_2_. Following 48 h incubation period, the cells were washed with PBS (pH 7.4) and incubated at 37°C for 30 min in pHrodo™ Green (Invitrogen, Thermo Fisher Scientific) staining solution (10 μl of pHrodo^TM^ Green AM added to 100 μl PowerLoad™ concentrate and finally diluted into 10 ml of PBS at pH 7.4). Next, the cells were washed twice and resuspended in PBS adjusted at pH 6.5, 7.4 or 8.0. After a 10 min incubation at room temperature, cells were washed twice again with PBS adjusted at the corresponding pH and fixed with 4% paraformaldehyde adjusted at pH 6.5, 7.4 or 8.0 for 10 min at room temperature (non-fixed cells were incubated in PBS adjusted at the corresponding pH). Then, the cells were washed twice again and incubated for an additional 10 min in PBS adjusted at pH 6.5, 7.4 or 8.0. Immediately afterwards, fluorescence was measured using a BioTek Synergy H1 microplate reader (Agilent Technologies) equipped with Gen5 software (bottom optics positioning, excitation: 500 nm, emission: 535 nm). All samples were prepared in triplicate.

### Quantum mechanics (QM) calculations

All calculations were performed with Gaussian16 Rev. B.01 software (55). Atomic coordinates for the QM calculations were extracted from experimental structures in the case of in **NN4_tC^O^2** and from the previously reported structure of **NN4** (10) (PDB 8BV6) and were relaxed by QM geometry optimization keeping always frozen intermolecular arrangements. To reduce noise only the methyl-capped (N9 purines, N1 pyrimidines and N3 for tC^O^) were considered. Geometry optimizations were done at the DFT level of theory using the B3LYP functional and the 6–311G(d) basis set, with a continuum representation of solvent as described by the PCM model (56,57). HOMO/LUMO energies where derived from the optimized geometries.

Binding energy calculations were performed at the B3LYP/6–311G(d) DFT level of theory, applying Grimme's strategy with Becke-Johnson damping D3-BJ (58) to correct for dispersion. Basis Set Superimposition Error (BSSE) was corrected through the Counterpoise correction method (59), as implemented in Gaussian.

### Fixed-cell fluorescence microscopy

For fixed-cell fluorescence microscopy experiments, HeLa cells (cultured in Dulbecco's Modified Eagle Medium supplemented with 10% fetal bovine serum) were seeded at a density of 50 000 cells per well on 24-well plates (Nunclon, Thermo Fisher Scientific) with 12 mm cover glasses on the well bottom and incubated at 37ºC in a humidified atmosphere with 5% CO_2_. Following overnight culture, the cells were transfected with the **NN4_tC^O^2** or the tC^O^2 Control oligonucleotides (500 nM doses) using Lipofectamine 2000 (Thermo Fisher Scientific) or left untreated (for the incubation with pHrodo). After a 20 h incubation period, the non-transfected cells were washed with PBS (pH 7.4) and incubated at 37ºC for 30 min in pHrodo™ Green (Invitrogen, Thermo Fisher Scientific) staining solution (10 μl of pHrodo™ Green AM added to 100 μl PowerLoad™ concentrate and finally diluted into 10 ml of PBS at pH 7.4). Next, all the cells were washed twice and resuspended in PBS adjusted at pH 6.5, 7.4 or 8.0. After a 10 min incubation at room temperature, cells were washed twice again with PBS adjusted at the corresponding pH and fixed with 4% paraformaldehyde adjusted at pH 6.5, 7.4 or 8.0 for 10 min at room temperature. Finally, cells were washed twice again and incubated for an additional 10 min in 5 μM DRAQ5 (Invitrogen, Thermo Fisher Scientific) in PBS adjusted at the corresponding pH. Next, the DRAQ5 staining solution was discarded, the cover slides were transferred to microscope slides (Avantar) with Fluoromount-G (Electron Microscopy Sciences) and samples were incubated overnight at 4°C prior to visualization.

Fluorescence of the pHrodo™ Green and the oligonucleotides was observed using a SPE confocal microscope (Leica) equipped with LAS AF software. tC^O^2 fluorescence (emission wavelength of 417–477 nm) was visualized using a 405 nm laser diode [20% intensity, 1050 Gain]; pHrodo Green fluorescence (emission wavelength of 520–560 nm) was visualized using a 488 nm laser diode [5% intensity, 1050 Gain] and DRAQ5 fluorescence (emission wavelength of 663–737 nm) was visualized using a 635 nm laser diode [20% intensity, 1050 Gain]). Images were analyzed using ImageJ software (NIH Image).

## Results

### tC^O^ can enhance i-motif thermal stability

**Figure 2.**
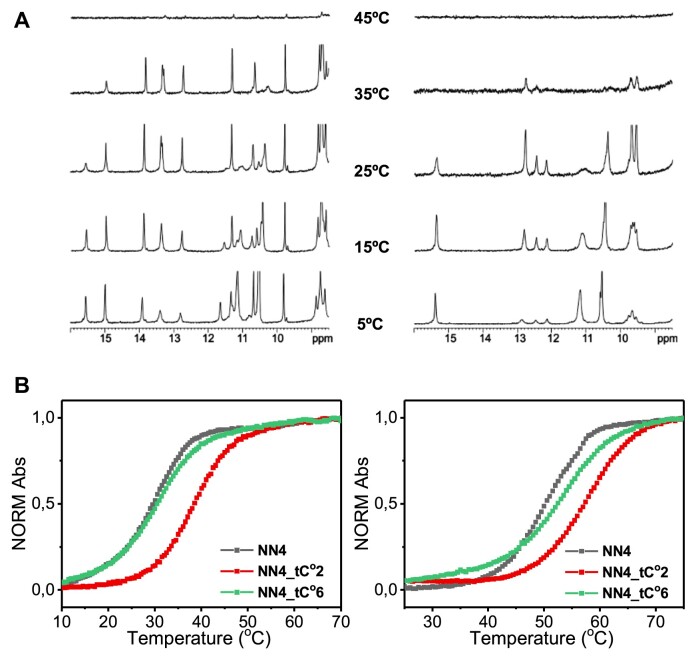
(**A**) ^1^H-NMR spectra at different temperatures of NN4_tC^O^2 (left) and NN4_tC^O^6 (right) at pH 7, 10 mM phosphate buffer, [oligonucleotide] = 1 mM. (**B**) UV-melting curves of NN4, NN4_tC^O^2 and NN4_tC^O^6 at pH 7 (left) and pH 5 (right). 25 mM phosphate buffer, [oligonucleotide] = 2 μM.

I-motif formation and thermal stability of the different sequences shown in Table 1 were monitored by NMR and UV-melting experiments. In all cases, ^1^H-NMR spectra indicate the formation of stable i-motif structures at neutral pH (Figure 2A). Similar signal pattern in the exchangeable proton region as that of the NMR spectra of unmodified **NN4** is observed for **NN4_tC^O^2** and **NN4_tC^O^6**, suggesting the formation of very similar structures, including hemiprotonated, C:C^+^ or tC^O^:C^+^ (15–16 ppm), and neutral G:C or G:tC^O^ base-pairs (12–14 ppm). Imino signals of C:C^+^ base pairs for the sequence **NN4_tC^O^2** are observed at higher temperatures than those of **NN4** (32) and **NN4_tC^O^6** (Figure 2A), indicating a considerably enhanced thermal stability in this sequence. This is confirmed by UV-monitored melting experiments (Figure 2B). The stabilizing effect of incorporating tC^O^ is sequence-specific. Whereas at position 6 the change in melting temperature is small, tC^O^ at position 2 provokes a large *T*_m_ increase. Interestingly, the effect is much more pronounced at neutral pH, where **NN4_tC^O^2** exhibits a Δ*T*_m_ of almost 10ºC (Table 1). NMR, CD and UV melting curves indicate that the structure and stability of **NN4_tC^O^2** are very similar in presence of Na^+^ or K^+^ counterions (Supplementary Figure S1).

### The structures exhibit different NMR and CD spectra at neutral and acidic pH

**Figure 3.**
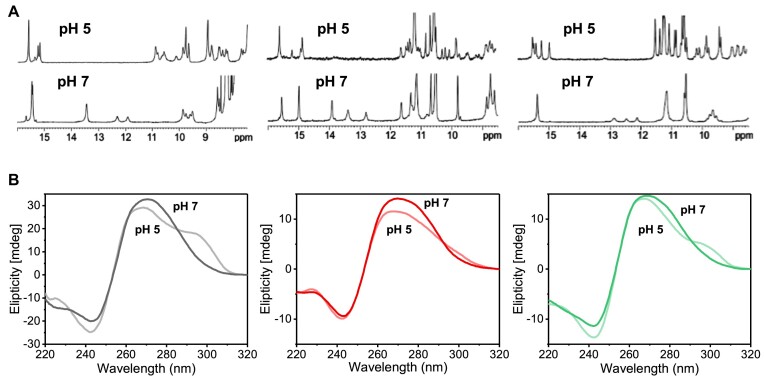
(**A**) ^1^H NMR spectra of NN4, (left) NN4_tC^O^2 (center) and NN4_tC^O^6 (right) at pH 5 and 7 at 5ºC. 10 mM phosphate buffer, [oligonucleotide] = 1 mM. (**B**) CD spectra of NN4, (left) NN4_tC^O^2 (center) and NN4_tC^O^6 (right) at pH 5 and 7 at 5ºC. 25 mM phosphate buffer, [oligonucleotide] = 2 μM.

^1^H-NMR spectra recorded at pH 5 (Figure 3A) indicate that tC^O^-containing sequences fold into alternative i-motif structures under acidic pH conditions. Spectra recorded under these conditions show more signals in the 15–16 ppm and 11–12 ppm regions. In addition, imino signals in the 12–14 ppm region are not observed, indicating that the G:C base pairs are not formed.

Substantial changes are also observed in the CD spectra of **NN4_tC^O^2** and **NN4_tC^O^6** at different pH (Figure 3B). At neutral pH CD spectra exhibit a minimum at ∼240 nm and a maximum at ∼270 nm. However, at pH 5 a slight decrease of intensity at 270 nm is observed, together with the formation of an additional band near 300 nm. These data suggest that **NN4_tC^O^2** and **NN4_tC^O^6** may undergo a similar conformational transition to that observed in **NN4** at acidic pH (10).

### NN4_tC^O^2 and NN4_tC^O^6 exhibit different fluorescence behavior

In order to assess the tC^O^ fluorescence under the different structural contexts provided by **NN4_tC^O^2** and **NN4_tC^O^6**, their excitation and emission spectra were recorded at different temperatures and pH values (Supplementary Figure S2). As expected for tC^O^-containing sequences, maximum absorption bands are found at 370 nm and maximum emission bands at 450 nm. Most interestingly, a dramatic difference between the fluorescence signals for the two modified sequences is observed at low temperature and neutral pH. Whereas the fluorescence signal is heavily quenched for the sequence **NN4_tC^O^2**, the effect is much less pronounced for the sequence **NN4_tC^O^6**. Remarkably, the recovery of the fluorescence signal of **NN4_tC^O^2** upon temperature increase takes place at approximately 35°C, matching the melting temperature obtained by UV spectroscopy (Figure 2B), and supporting the use of fluorescence to trace the folding/unfolding transition of **NN4_tC^O^2**. A similar quenching profile is observed at acidic pH, although the fluorescence recovery occurs at higher temperature values (*T*_m_ ∼ 55ºC). In the case of **NN4_tC^O^6**, quenching is more pronounced at acidic than at neutral pH, suggesting a different chemical environment of tC^O^ residue under these experimental conditions.

### tC° fluorescence behavior in B-DNA

As previously described in the literature (31). tC^O^ shows a notably low quenching of the emitted fluorescence signal when base paired to guanine in a DNA duplex. In order to assess the difference in quenching displayed by tC^O^ in an i-motif and a duplex context, fluorescence experiments were recorded for **NN4_tC^O^2** in the presence of its complementary strand, d(ACGGAACGGAAAAACGGAACGG), at different temperatures. As expected, the resulting duplex exhibits a very low quenching of the fluorescence signal at all temperatures (see Supplementary Figure S3).

### Structural determination of NN4_tC^O^2 at neutral pH

**Figure 4.**
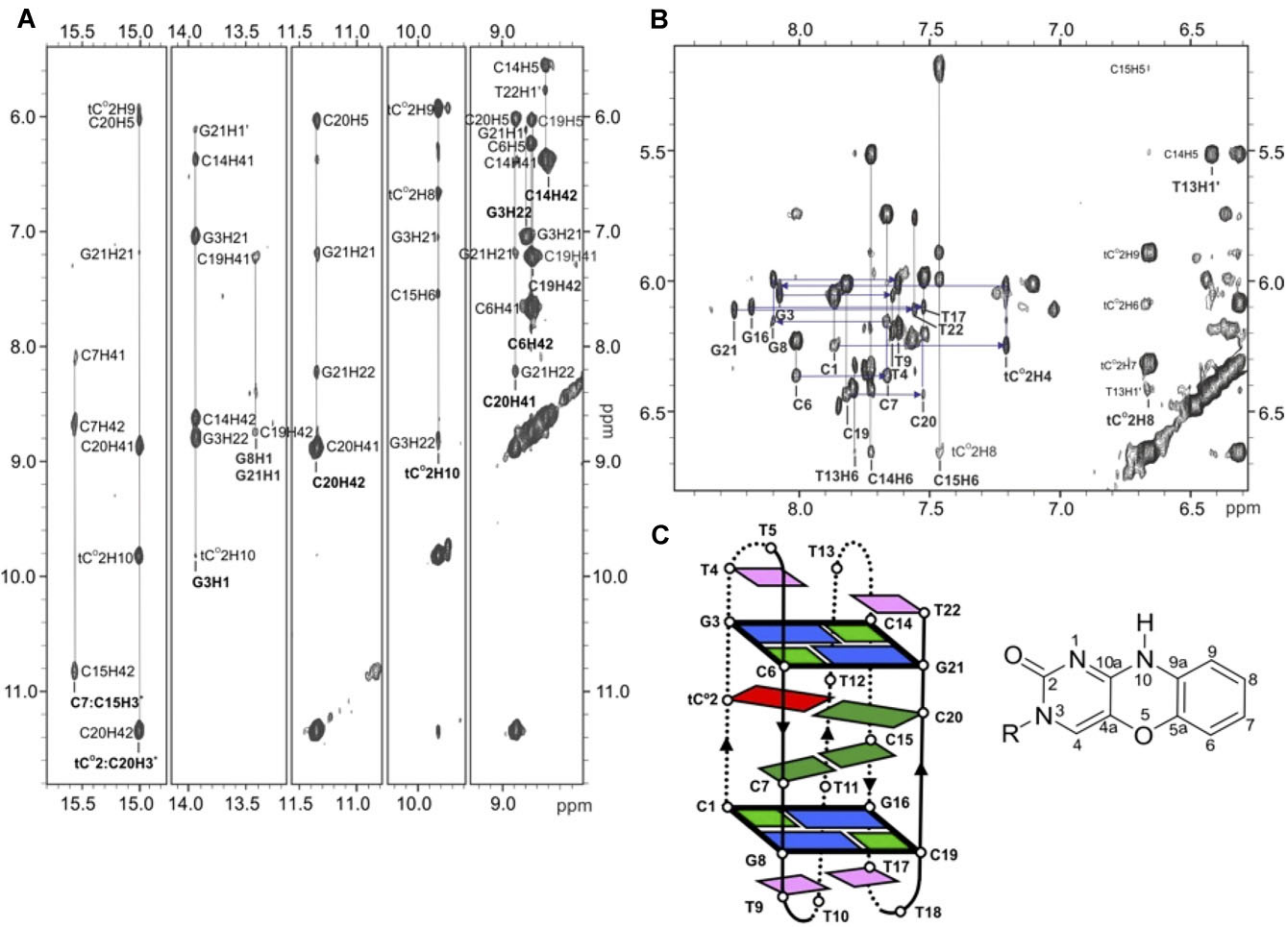
Exchangeable (**A**) and non-exchangeable (**B**) protons region of the NOESY spectrum (150 ms) of NN4_tC^O^2 at pH 7 and *T* = 5ºC. Numbering scheme of tC^O^ residue (**C**). 10 mM phosphate buffer, H_2_O/D_2_O 90:10, [oligonucleotide]= 1 mM.

To get more insight in the structural bases of the sequence-specific thermal and pH stabilization induced by tC^O^, as well as the distinctive tC^O^ fluorescent behavior in different environments, the structural characterization of **NN4_tC^O^2** and **NN4_tC^O^6** structures at acidic and neutral conditions was undertaken by NMR methods. We focused first on **NN4_tC^O^2** at neutral pH, in which conditions the NMR spectra exhibit very well-dispersed narrow signals. The number of thymine Me-H6 and cytosine H5-H6 cross-peaks identified in the TOCSY spectrum, and the observation of a single aromatic spin system corresponding to the tC^O^2 residue are consistent with the formation of a single folded species. Those contacts involving tC^O^ were essential for determining the folding topology (Figure 4C) and completing the sequential assignment (see Figure 4 and Supplementary Figures S4, S5 and S6). As shown in Figure 4A, two expected imino proton signals corresponding to hemiprotonted C:C^+^ base pairs are observed at 15.57 and 15.01 ppm. The signal at 15.01 ppm was unequivocally assigned to the tC^O^2:C20^+^ base pair on the basis of its cross-peak with H10 and with two amino protons of C20 (see Figure 4C for tC^O^ numbering). The other imino signal was assigned to C7:C15^+^. In addition, four guanine imino signals can be observed in the Watson–Crick region (see Figure 4 and Supplementary Figure S4). The one at 13.93 ppm is the most intense and exhibits a stacking cross-peak with tC^O^2H10. Consequently, it was assigned to G3 which is base paired to C14. On the basis of the cross-peaks with some contacts with the corresponding base-paired cytosines, imino signals at 13.44 and 13.37 ppm were assigned to G8 (paired to C19) and G21 (paired to C6), respectively (see Supplementary Figure S4). The remaining signal at 12.82 ppm was assigned to G16, although no cross peak with C1 amino protons could be detected. Reciprocal G3–G21 and G8–G16 imino-H1’ contacts across the minor groove are observed (Figure 4 and Supplementary Figure S4), confirming the minor groove tetrad formation. Chemical shifts and a graphical view of the most relevant contacts are shown in Supplementary Tables S1, S2 and Supplementary Figure S7, respectively.

The solution structure of **NN4_tC^O^2** at pH 7 was calculated on the basis of 115 NOE-derived distance constraints (see details in Supplementary Table S3). The resulting ensemble of 10 structures is shown in Supplementary Figure S8. The structure is well-defined, with RMSD values <1 Å (see Supplementary Table S3 for calculation statistics). Dihedral torsion angles values are in general well-defined (see Supplementary Table S4), with glycosidic angles in the *anti* conformation. As found in similar structures (8,10,45), all sugar rings adopt an *S*-type conformation, predominantly in the *C2’-endo* region (Supplementary Table S5). Cytosine residues involved in C:C^+^ pairs mainly adopt a *C1’-exo* conformation.

**Figure 5.**
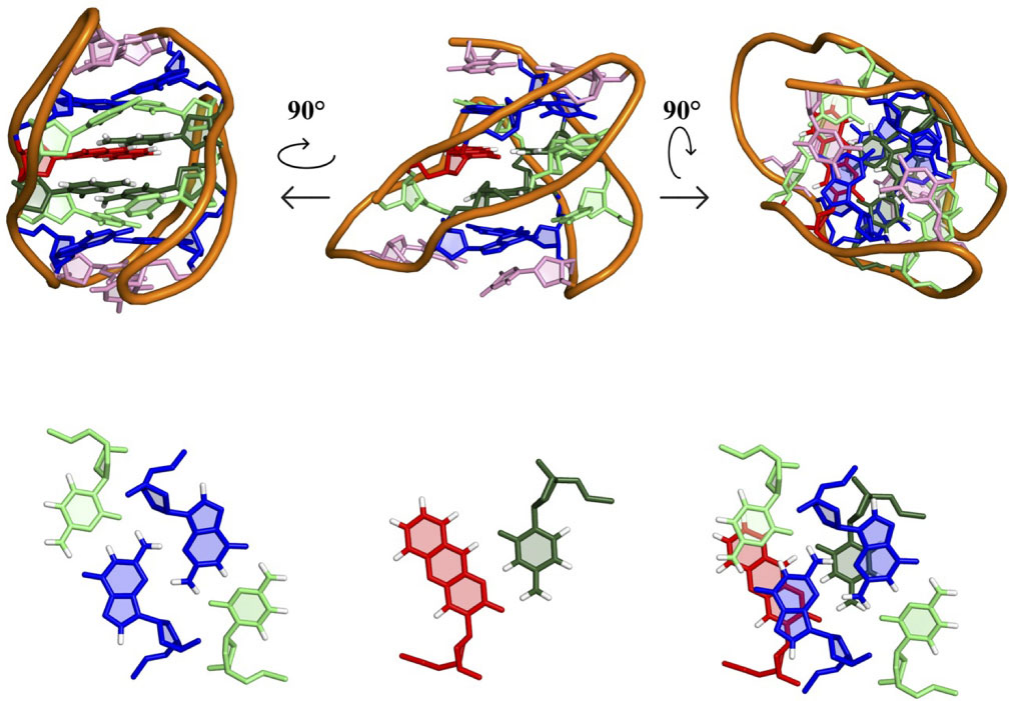
(Top) Different views of calculated structure of NN4_tC^O^2. (Bottom) Structural details of the G:C:G:C tetrads, tC^O^:C^+^ hemiprotonated base pair, and stacking of tC^O^:C^+^ and minor groove tetrad.

Overall, the structure of **NN4_tC^O^2** is very similar to the structure of unmodified sequence **NN4** (Supplementary Figure S9) (10). The core of the structure is formed by two hemiprotonated base pairs (one C:C^+^ and one tC^O^:C^+^) and two slipped G:C:G:C minor groove tetrads at both ends as capping elements (Figure 5). The tetrad G3:C14:G21:C6 is perfectly stacked on top of the tC^O^:C^+^ base pairs, burying almost completely the tC^O^ nucleobase (see Figure 5). The enhanced stacking interaction provided by the bigger size of tC^O^ versus a cytosine nucleobase is responsible for the remarkably enhanced stability exhibited by **NN4_tC^O^2** compared to NN4. Indeed, quantum-level calculations indicate a stacking interaction between the G:C base pair of the tetrad and tC^O^ to be about 3 kcal/mol stronger than with a normal cytosine (–14.0 kcal/mol for the fluorophore compared with –11.0 kcal/mol for cytosine) at the DFT level of theory (see Materials and methods).

### Structural characterization of NN4_tC^O^2 at acidic pH

NMR spectra of **NN4_tC^O^2** were acquired at different acidic conditions (pH 4, 5 and 6). Very little change is observed between pH 6 and 7 (see Supplementary Figure S10), indicating that folding pattern does not change within this pH range. However, significant spectral changes occur at pH 5 (see Supplementary Figure S11), including new imino proton signals in the C:C^+^ imino region, and up to 13 cytosine H5–H6 cross-peaks in the TOCSY spectrum. Also, two sets of tC^O^2 spin systems can be clearly identified. All this is consistent with the presence of two species in a slow equilibrium in the NMR time scale, being one of them that found at pH 7.

To characterize the second species, pH was lowered to 4. In these conditions, acidic species is more populated, although some signals corresponding to the neutral form are still observed. In the hemiprotonated imino region, four signals are observed: 15.65, 15.24, 14.95 and 14.79 ppm (see Supplementary Figure S12). The signal at 15.65 ppm exhibits cross-peaks with four pairs of amino protons that correspond to the C6:C14^+^ and C1:C19^+^ base pairs in the acidic form. The signal at 15.24 ppm corresponds to C7:C15^+^ base pair and the signal at 14.95 ppm corresponds to the tC^O^2:C20^+^ base pair, also in the acidic species. The signal at 14.79 corresponds to the tC^O^2:C20^+^ base pair of the neutral form, still observed as a minor species at this pH. The assignment of cytosine residues of the acidic form could be accomplished by following the contacts of C20 and C14 with tC^O^2 (C20H3^+^-tC^O^2H10, tC^O^2H8-C14H6) (see Supplementary Figure S13). C1 residue could be assigned on the basis of the characteristic C14/C1 H42-CH2’/H2’ cross-peaks between 3′ sides of intercalated bases. C7H1’-C1H1’ allowed distinguishing between C7 and C15. Two G:T imino-imino protons cross-peaks were clearly observed (see Supplementary Figure S12). According to the observed contacts between guanine imino and cytosine amino protons, these two pairs could be assigned to G8:T18 and G21:T5 base pairs. The observation of NOE contacts between the aromatic system of tC^O^ and C14 (tC^O^H8-C14H6 and tC^O^H9-C14H5), indicates that they face their 5′-5′ sides through the major groove at pH 4. Moreover, the highly upshifted chemical shift of C14H5 (5.11 ppm), suggests that C14 in the acidic form occupies the equivalent position as C15 in the neutral species.

We can conclude that **NN4_tC^O^2** exhibits a similar pH-dependent conformational equilibrium between two i-motif species than the unmodified sequence **NN4** (10), the acidic species being in both cases stabilized by the formation of four hemiprotonated base pairs. However, in **NN4_tC^O^2** not all the G:T base pairs expected for the formation of two capping G:T:G:T tetrads were observed, specifically those that required the involvement of thymine residues from the central loop (G3:T13 and G16:T10). The presence of signals from the neutral species at low pH indicates that the neutral structure of **NN4_tC^O^2** is the predominant form in a more ample range of pH than in the case of **NN4**. The better stacking of the fluorophore compared with cytosine justifies this stabilization (see above).

### Structural characterization of NN4_tC^O^6 at neutral and acidic pH

At neutral pH, **NN4_tC^O^6** NMR spectra exhibit two nearly overlapped imino signals at 15.40 and 15.38 ppm with cross-peaks with two amino protons pairs, indicating the formation of two C:C^+^ base pairs. Seven H5–H6 cross-peaks, ten Me-H6 cross-peaks and a unique aromatic spin system corresponding to tC^O^ are observed in the TOCSY spectrum, indicating the formation of a single species at these conditions. Stacking connections H6/H8-H2’/H2’ can be easily stablished for the four 5′-CCGT-3′ tracts. The presence of tC^O^6 residue provides sequential C7H6-tC^O^6H2’/H2’ and tC^O^6H4-T5H1’/H2’/H2’ cross-peaks and some contacts between tC^O^6 residue and a cytosine not involved in hemiprotonated base-pairs. Additionally, the presence of some cross-peaks involving thymine connecting loop indicates that this loop is located close to one of the i-motif major grooves and confirms the global folding (see Supplementary Figure S14 and S15 for detailed assignment). Although the characteristic G:C Watson–Crick cross-peaks are weak, the signals between 12–13 ppm could be assigned to G3, G8, G16 and G21, according to their cross-peaks with H5 of the stacking cytosines C2, C7, C15 and C20. Although no cross-peak was observed between any guanine imino proton and tC^O^6H10, the chemical shift of H10 (11.17 ppm) indicates its implication in hydrogen bond formation. Altogether, the experimental data at neutral pH are consistent with the formation of the i-motif structure shown in Figure 1B, right capped by G:C W-C base pairs in which tC^O^ unit is located in one of the tetrads.

At lower pH, two sets of aromatic signals corresponding to tC^O^ residues are observed. According to the signal intensities, the acidic specie is the major one. In this species, a number of NOEs involving tC^O^ protons show that a tC^O^:C^+^ base-pair is formed and it is located in the center of the C-stack (i.e. H10 proton exhibit characteristic amino-H2’/H2’ cross-peaks between 3′-3′ intercalated base pairs alongside the major groove). Although complete sequential assignment could not be carried out due to severe signal overlapping in the aromatic region, all the residues involved in the C:C^+^ stack and the tetrads could be identified (see Supplementary Figures S16 for details). The experimental data are consistent with the formation of a more elongated i-motif structure, analogous to that found for **NN4** at acidic pH.

### Theorical calculations of HOMO–LUMO energies

To gain further insight into the observed photophysical behavior of tC^O^ in the contexts of minor groove stabilized i-motifs, the HOMO LUMO energies of different nucleobases and nucleosides dimers and tetramers were calculated (see Supplementary Figure S17). First evident result is that the HOMO/LUMO gap is reduced when the fluorophore is protonated (3.61 eV for tC^O+^ compared with 4.10 eV for tC^O^, which can explain the small red-shifted peak at low pH (see for example Supplementary Figure S2) that will correspond to a residual percentage of the protonated fluorophore (similar shifts were detected by Karimi *et al.* (60)). The HOMO/LUMO of the fluorophore are within the range of all the different neighboring bases or base combinations (see Supplementary Table S7 and Supplementary Figure S17), explaining the great fluorescent properties of tC^O^. The exception is protonated cytosine, where both frontier orbitals are displaced towards lower energy values suggesting that Photoinduced Electron Transfer (PET) (61,62) can be expected when the fluorophore is very close to a protonated cytosine, leading then to strong quenching.

### pH-driven transitions monitored by CD and fluorescence

**Figure 6.**
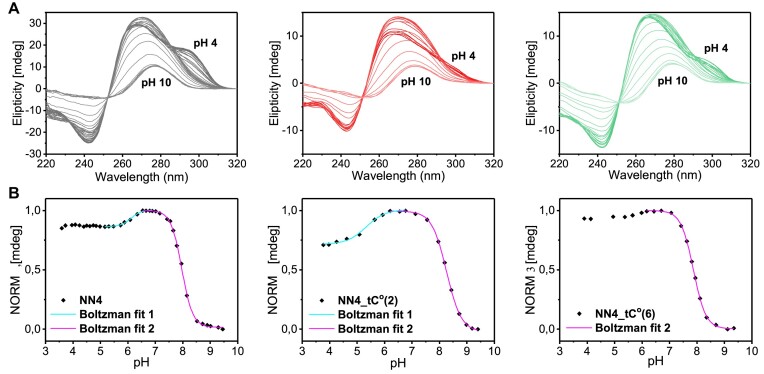
(**A**) CD spectra of NN4 (left), NN4_tC^O^2 (center) and NN4_tC^O^6 (right) at 5ºC and different pH values. 25 mM phosphate buffer, [oligonucleotide] = 2.0 μM. (**B**) CD-monitored pH titration curves at 265 nm of NN4 (left), NN4_tC^O^2 (center) and NN4_tC^O^) (right) at 5 ºC. Boltzmann fit represented in magenta for the denaturation of the structures (pH_T2_) and in cyan for the equilibrium between species (pH_T1_).

The conformational transitions between the different structures adopted by **NN4_tC^O^2** and **NN4_tC^O^6** were monitored by CD and fluorescence. The two isosbestic points at 250 and 295 nm in the CD spectra are indicative of the existence of two structural transitions involving three species (see Figure 6A). CD pH-titration curves were obtained by plotting maximum ellipticity values at two different wavelengths, 265 and 295 nm (see Figure 6B and Supplementary Figure S18, respectively). pH_T_ values are shown in Table 2. In the case of **NN4_tC^O^2** at 265 nm, a first transition is observed with a pH_T1_ of 5.4 (cyan line) which corresponds to the interconversion between the acidic and neutral i-motif structures. The second transition (magenta line) presents pH_T2_ value of 8.3 and corresponds to the denaturation of the structure upon deprotonation of cytosine residues. At this wavelength, this is the only transition exhibiting a substantial ellipticity change for **NN4_tC^O^6**, with a pH_T_ value of 7.9. Interestingly, the neutral form of **NN4_tC^O^2** is the major species in a wider pH range than the other two sequences.

**Table 2.** pH_T_ values calculated from CD and fluorescence experiments for NN4, NN4_tC^O^2 and NN4_tC^O^6. 25 mM phosphate buffer, [oligonucleotide] = 2 μM (CD), 0.2 μM (fluorescence)

NAME	CD 265 nm		CD 295 nm		Fluorescence
	pH_T1_	pH_T2_	pH_T1_	pH_T2_	pH_T_
**NN4**	6.1 ± 0.1	8.0 ± 0.1	6.1 ± 0.1	7.9 ± 0.1	-
**NN4_tC^O^2**	5.4 ± 0.1	8.3 ± 0.1	-	8.3 ± 0.1	8.0 ± 0.1
**NN4_tC^O^6**	-	7.9 ± 0.1	6.0 ± 0.1	7.8 ± 0.1	6.3 ± 0.1

Transitions can be also monitored at 295 nm (Supplementary Figure S19). In this case, the only transition that exhibits a substantial ellipticity decrease for **NN4_tC^O^2** corresponds to the i-motif denaturation (pH_T2_ 8.3). However, in the case of **NN4_tC^O^6** the two transitions can be clearly detected (pH_T1_ 6.0 and pH_T2_ 7.8). At this wavelength, another transition at around pH 4, corresponding to the denaturation of the acidic species due to the complete protonation of cytosines, can be observed in the three cases (Supplementary Figure S19). A schematic view of the different transitions involved is shown in Supplementary Figure S20.

**Figure 7.**
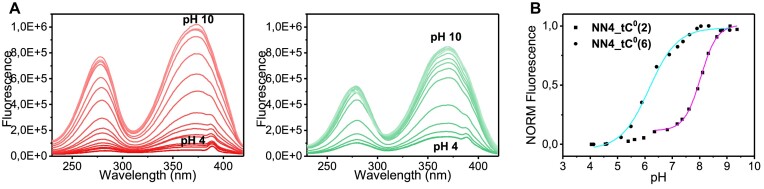
(**A**) Fluorescence excitation spectra recorded at different pH values for NN4_tC^O^2 (left) and NN4_tC^O^6 (center). (**B**) Fluorescence-monitored pH-titration curves for NN4_tC^O^2 and NN4_tC^O^6 (right). Boltzmann fit represented in cyan for the sequence NN4_tC^O^6 and in magenta for the sequence for NN4_tC^O^2. 5ºC. 25 mM phosphate buffer, [oligonucleotide] = 0.2 μM.

Fluorescence pH-titration experiments were also carried out recording excitation spectra over a range of pH from 3.5 to 9.5 (Figure 7A). Under acidic conditions both sequences exhibit a high quenching of the fluorescence signal, whereas fluorescence signal gradually increases at higher pH values. Boltzmann fit of the pH-dependence of fluorescence at 370 (Figure 7B) allowed the determination of pH_T_ values for these transitions (see Table 2). In contrast to CD experiments, only one transition exhibits a dramatic fluorescence change. Interestingly, fluorescence recovery occurs at lower pH values for **NN4_tC^O^6** than for **NN4_tC^O^2**. Fluorescence changes can be rationalized on the basis of the chemical environment surrounding tC^O^ and the structural features of the different species involved in the equilibria. In the case of **NN4_tC^O^2** (Figure 7B, magenta line), tC^O^ is involved in hemiprotonated base pairs and stacking interactions that are maintained in the neutral and acidic structures. Therefore, the only important change in the fluorophore chemical environment occurs upon i-motif unfolding. However, the transition between the acidic and neutral i-motif structures of **NN4_tC^O^6** (cyan line) implies a dramatic change in the tC^O^ chemical environment, since tC^O^ forms a hemiprotonated pair in the acidic form and a G:C pair in the neutral form. Thus, we must conclude that fluorescence experiments are only sensitive to the first CD-monitored transition (pH_T1_) in the case of **NN4_tC^O^6**, and the second one (pH_T2_) for **NN4_tC^O^2**. In both cases, the particular pH_T_ values determined by CD or fluorescence are very similar.

To gain further insight into the impact of the chemical environment on the fluorescence behavior of tC^O^, we prepared a control oligonucleotide containing tC^O^ and unable to fold into an i-motif. This control oligonucleotide (**controlNN4_tC^O^2)** has a sequence very similar to **NN4_tC^O^2**, but the order of nucleotides in the tetrad is altered in a way that prevents the formation of the minor groove tetrad. NMR spectra clearly indicate that this permutation leads to a complete destabilization of the i-motif at all pHs (Supplementary Figure S21). However, the fluorescence spectra still show a pH and temperature dependence (Supplementary Figure S21). These results, together with the theoretical calculations described earlier, strongly suggest that the tC^O^ fluorescence is influenced by the protonation state of its surroundings.

### I-motif folding monitored *in cellulo* by fluorescence microscopy

Due to the good results obtained for **NN4_tC^O^2** in terms of stability and fluorescence behavior, we considered this sequence a strong candidate to test the visualization of the mini i-motif structure *in cellulo*.

**Figure 8.**
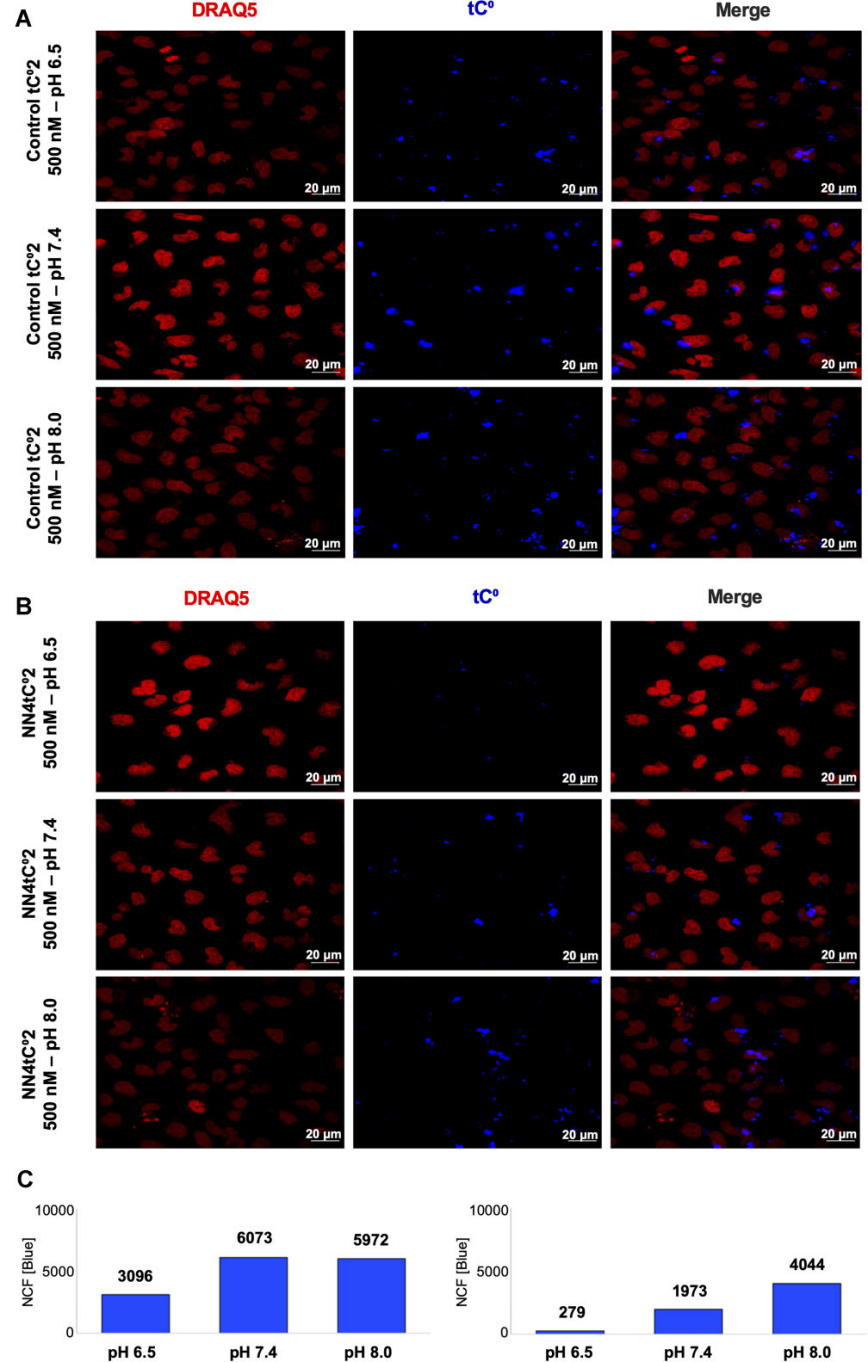
Sequence of images showing the fluorescence signal of controlNN4_tC^O^2 (**A**) and NN4_tC^O^2 (**B**) of transfected Hela cells at different pH values. (**C**) Graphical representation of pH-dependent variation of fluorescence for controlNN4_tC^O^2 (left) and NN4_tC^O^2 (right). Images were recorded 20 h after transfection. Note that there is an increase in quenching by a factor of 2 upon decreasing the pH from 8 to 6.5 in the control oligo (left panels), where the same change in pH leads to 20 times more quenching in the case of NN4_tC^O^2.

In order to assess if the formation of mini i-motif structures is feasible in the cell, fixed-cell fluorescence microscopy experiments were carried out. HeLa cells (incubated at 37 ºC) were transfected with **NN4_tC^O^2** and the control oligonucleotide **controlNN4_tC^O^2** in the presence of Lipofectamine 2000 ([oligonucleotide] = 500 nM). Experiments were carried out at three different pH values in order to visualize the pH-dependent i-motif formation in cells. Cell nuclei were stained with DRAQ5 dye (far-red emision) and pHrodo^TM^ Green was used to monitor intracellular pH changes upon changing cell media (Supplementary Figure S22). The pHrodo^TM^ Green fluorescence exhibited an inverse correlation with extracellular pH, showing a three-fold increase from pH 7.4 to pH 6.5 and a 2-fold decrease from pH 7.4 to pH 8.0 in fixed cells. This suggests a similar intracellular and extracellular pH (Supplementary Figure S23). As shown in Figure 8 and Supplementary Figures S24–S26, cells transfected with **controlNN4_tC^O^2** displayed a comparable blue fluorescence intensity at the three pH values, with only a slight reduction at acidic pH (see quantification in Figure 8). These findings clearly indicate that the negative control remains unfolded inside the cells and the pH-dependent changes in fluorescence are those expected from a partial protonation of the fluorophore (see above). In contrast, substantial changes in blue fluorescence intensity at different pH levels were observed in cells transfected with **NN4_tC^O^2**. The fluorescence changes correlate well with the expected folding/unfolding transition of **NN4_tC^O^2**. At physiological pH, the decrease in fluorescence compared to the negative control (∼33%), aligns with the thermal denaturation data, which indicated a population of around 50% of folded species at 37ºC and pH 7. Acidification of the media induces the folding of **NN4_tC^O^2**, as indicated by the quenching of fluorescence emission, whereas rising pH provokes the denaturation of the motif and, consequently, an increase in fluorescence. Considering that **NN4_tC^O^2** is mainly unfolded at pH 8 and 37ºC, the quenching of the fluorescence signal at pH 7.4 is around 50%, and higher than 90% at pH 6.5.

## Discussion

**NN4** and its analogs are able to adopt two different i-motif structures, making it a unique case of conformational switch between two different i-motifs (10). The relative stability of the two species depends on the environmental conditions (pH and temperature), and it is determined by cytosines’ protonation states. Since the coexistence of neutral and protonated cytosines forming either C:G or C:C^+^ base pair is a key feature of this system, the idea of exploring fluorescent cytosine analogs with similar base-paring properties emerges naturally. tC^O^ or related cytidine analogs (tC and DMAC), which are able to base pair with guanines or cytosines, meet these requirements. Although a number of studies have reported the use of tC^O^ in i-motifs, they have not explored those i-motifs stable at physiological pH. Reilly *et al.* studied a 20-mer variant of the C-rich strand of the C-MYC promoter with tC^O^ substitutions in C:C^+^ base pair and in the loops. At acidic pH, these substitutions always reduced the thermal stability, and showed very little change on the pH_T_ (23). Similar effects were observed by Bielecka et al. with i-motif forming sequences based on RET proto-oncogene (24,27). Despite the interest in this nucleobase analog, no detailed structural studies of i-motifs containing tC^O^ had been carried out until now.

In this study, we have exploited the peculiar properties of **NN4** to investigate the impact of tC^O^ on i-motifs in their neutral and hemiprotonated states. NMR analysis reveals that the primary characteristics of **NN4**’s neutral and acidic structures are retained in the modified i-motifs. In the case of **NN4_tC^O^6**, the neutral and acidic structures keep similar thermal stabilities as in the unmodified ones. Furthermore, transition between the different species occur at similar pH_T_ values. However, in the case of **NN4_tC^O^2**, significant improvements in thermal stability are observed, being more pronounced in the neutral structure. Three-dimensional structural determination by NMR methods clearly indicates that this effect arises from the critical interaction between tC^O^:C^+^ base pairs and G:C:G:C minor groove tetrads, which is likely to be related to a better stacking (see results above). The NMR data also suggest that the acidic structures is similar to the unmodified **NN4**, leading to the conclusion that the interaction of tC^O^:C^+^ is more favorable with G:C:G:C than that with G:T:G:T tetrads. This enhanced stability is also reflected in the transitional pH_T_s between the acidic and unfolded species, with an increase of one pH unit in the range of pH where the neutral structure is the major species. The capacity of G:C:G:C minor groove tetrads to stabilize neighbouring positively charged cytosines and to strongly increase their p*K*_a_ has been reported in our previous study of **NN4**. This favourable interaction is due to strong Pi-cation interactions, as shown by Poisson–Boltzman calculations, which fairly reproduce the experimentally observed p*K*_a_ shifts (10). We demonstrate here that the robust pH and temperature stabilization induced by MGTs is even more pronounced when the neighbouring base pair is tC^O^:C^+^ instead of C:C^+^. The favorable interaction between the large aromatic system of the nucleobase analog tC^O^ with the nearby G:C:G:C tetrad in **NN4_tC^O^2** (related to improved stacking interactions; around 3 kcal/mol from DFT calculations) results in a more stable i-motif in which the tC^O^:C^+^ base pair remains protonated at an unusually high pH.

It is interesting to note that not all the transitions occurring in **NN4** and its modified analogs lead to changes in fluorescence. Again, this effect can be explained on the light of the structural analysis. In the case of **NN4_tC^O^6**, tC^O^ is involved in a tC^O^:G base-pair in the capping tetrad at neutral conditions. The unfolding of this structure does not provoke a dramatic change in the cationic environment around tC^O^ and, consequently, not large changes in fluorescence quenching are expected upon melting at neutral pH. On the contrary, melting can lead to a dramatic effect at acidic pH, where tC^O^ is placed in a cationic environment (paired with a protonated cytosine) in the folded structure, but not in the unfolded state. In contrast, pH modification does not alter the microenvironment of the fluorophore in **NN4_tC^O^2**, which in the folded state is always paired with a protonated cytosine. As a result, the dramatic gain in fluorescence upon unfolding should be the same irrespective of the pH.

It is clear that the formation of the tC^O^:C^+^ pairs is the main responsible of the fluorescence quenching detected here, but the origin of this effect is not so evident. One possibility is that a proton transfer from the cytosine to the fluorophore leading a tC^O+^:C pair which would lead to non-radiative energy dissipation due to protonation/deprotonation dynamics (63). However, accurate QM calculations (see results), the small magnitude of the red-shifted peak, and the sizeable p*K*_a_ difference between C and tC^O^ (more than 2 units) argue against this idea, suggesting that the proton is mainly attached to the cytosine. Furthermore, fluorescence spectra of the control oligonucleotide which cannot form well-defined structure (even a certain amount of tC^O^:C^+^ could be expected) show a temperature dependent quenching at acidic pH, but much lower than that found for oligos able to form stable i-motif based structures. Second, and most likely the real reason is the presence of photoinduced electron transfer mechanism (PET) related to the frontier orbitals of the neutral fluorophore and the protonated cytosine as discussed in previous work (61), which would lead to a dramatic and structure-dependent quenching.

The spectroscopic properties of MGT stabilized i-motifs containing tC^O^ nucleobases are very interesting for a number of applications. Although a number molecular pH sensors (28,64,65) and other dynamical pH-dependent nanodevices (66–68) based on i-motifs have been proposed, the large thermal and pH stability conferred by the minor groove tetrads makes **NN4_tC^O^2** a useful sensor in a range of pHs inaccessible to other i-motif based devices. As a consequence, tC^O^ fluorescence emission is highly quenched at neutral pH, in contrast to that occurring when this oligonucleotide is unfolded or hybridized with its complementary sequence. Thus, **NN4_tC^O^2** is well suited to distinguish i-motif from duplex formation by fluorescence techniques. Moreover, since tC^O^ does not provoke large changes on the surface of the i-motif, this modification should not affect i-motif recognition by proteins and small ligands. Consequently, **NN4_tC^O^2** and related sequences might be used to detect proteins or ligands able to unfold i-motifs.

The combination of good fluorescence properties and enhanced thermal and pH stability makes **NN4_tC^O^2** and related sequences excellent tools to monitor i-motif formation in physiological conditions *in vitro* and inside the cell. We have shown here that changes in tC^O^ fluorescence can be used to visualize pH-induced folded/unfolded transitions. When tC^O^ is involved in tC^O^:C^+^ base pairs next to minor groove tetrads, it induces minimal distortion in the three-dimensional structure, making this system a valuable tool for studying i-motif/protein recognition. In many cases, whether a particular protein binds the i-motif structure or the unfolded sequence is cause of debate. Changes in tC° fluorescence in minimally distorted i-motifs may offer a more effective strategy for detecting protein-induced unfolding than incorporating large, bulky groups at the sides of the i-motif, as required in FRET experiments (69).

The particular i-motif structure formed by **NN4** and its analogues is interesting because of the prevalence of these sequences in the human genome (8). However, the strategy proposed here is not limited to this particular sequence family. Other biologically relevant i-motif forming sequences can be adapted by introducing a minor groove tetrads as capping element, as shown recently in the stabilization of i-motif/duplex junctions (45).

The occurrence of the i-motif in the cell and its role in biological processes remain controversial (18,69–71). The results described in this study provide a new tool for deciphering the potential functions of this intriguing DNA motif.

## Supplementary Material

gkae106_Supplemental_File

## Data Availability

Coordinates are deposited in the PDB data bank (code 80FC). The NMR data are deposited in BMRB under the code 34798. Raw data will be shared on request to the corresponding/first authors.

## References

[1] Gehring K. , Leroy J.L., Guéron M. A tetrameric DNA structure with protonated cytosine-cytosine base pairs. Nature. 1993; 363:561–565. 8389423 10.1038/363561a0

[2] Abou Assi H. , Garavís M., González C., Damha M.J. i-motif DNA: structural features and significance to cell biology. Nucleic Acids Res. 2018; 46:8038–8056. 30124962 10.1093/nar/gky735 PMC6144788

[3] Benabou S. , Aviñó A., Eritja R., González C., Gargallo R. Fundamental aspects of the nucleic acid i-motif structures. RSC Adv. 2014; 4:26956–26980.

[4] Day H.A. , Pavlou P., Waller Z.A.E. I-motif DNA: structure, stability and targeting with ligands. Bioorg. Med. Chem. 2014; 22:4407–4418. 24957878 10.1016/j.bmc.2014.05.047

[5] Brazier J.A. , Shah A., Brown G.D. I-motif formation in gene promoters: unusually stable formation in sequences complementary to known G-quadruplexes. Chem. Commun. 2012; 48:10739. 10.1039/c2cc30863k 23012694

[6] Fleming A.M. , Ding Y., Rogers R.A., Zhu J., Zhu J., Burton A.D., Carlisle C.B., Burrows C.J. 4n-1 Is a “sweet spot” in DNA i-motif folding of 2′-deoxycytidine homopolymers. J. Am. Chem. Soc. 2017; 139:4682–4689. 28290680 10.1021/jacs.6b10117

[7] Wright E.P. , Huppert J.L., Waller Z.A.E. Identification of multiple genomic DNA sequences which form i-motif structures at neutral pH. Nucleic Acids Res. 2017; 45:2951–2959. 28180276 10.1093/nar/gkx090 PMC5605235

[8] Mir B. , Serrano I., Buitrago D., Orozco M., Escaja N., González C. Prevalent sequences in the Human genome can form mini i-motif structures at physiological pH. J. Am. Chem. Soc. 2017; 139:13985–13988. 28933543 10.1021/jacs.7b07383

[9] Cheng M. , Qiu D., Tamon L., Ištvánková E., Víšková P., Amrane S., Guédin A., Chen J., Lacroix L., Ju H. et al. Thermal and pH stabilities of i-DNA: confronting in vitro experiments with models and In-cell NMR data. Angew. Chem. Int. Ed. 2021; 60:10286–10294. 10.1002/anie.202016801 33605024

[10] Serrano-Chacón I. , Mir B., Cupellini L., Colizzi F., Orozco M., Escaja N., González C. pH-dependent capping interactions induce large-scale structural transitions in i-motifs. J. Am. Chem. Soc. 2023; 145:3696–3705. 36745195 10.1021/jacs.2c13043 PMC9936585

[11] Belmonte-Reche E. , Morales J.C. G4-iM grinder: when size and frequency matter. G-Quadruplex, i-motif and higher order structure search and analysis tool. NAR Genomics Bioinformatics. 2019; 2:lqz005. 33575559 10.1093/nargab/lqz005 PMC7671307

[12] Kang H.J. , Kendrick S., Hecht S.M., Hurley L.H. The transcriptional complex between the BCL2 i-motif and hnRNP LL is a molecular switch for control of gene expression that can be modulated by small molecules. J. Am. Chem. Soc. 2014; 136:4172–4185. 24559432 10.1021/ja4109352 PMC3985447

[13] Kendrick S. , Kang H.J., Alam M.P., Madathil M.M., Agrawal P., Gokhale V., Yang D., Hecht S.M., Hurley L.H. The dynamic character of the BCL2 promoter i-motif provides a mechanism for modulation of gene expression by compounds that bind selectively to the alternative DNA hairpin structure. J. Am. Chem. Soc. 2014; 136:4161–4171. 24559410 10.1021/ja410934b PMC3985915

[14] Kaiser C.E. , Van Ert N.A., Agrawal P., Chawla R., Yang D., Hurley L.H. Insight into the complexity of the i-motif and G-quadruplex DNA structures formed in the KRAS promoter and subsequent drug-induced gene repression. J. Am. Chem. Soc. 2017; 139:8522–8536. 28570076 10.1021/jacs.7b02046 PMC5978000

[15] Takahashi S. , Brazier J.A., Sugimoto N. Topological impact of noncanonical DNA structures on Klenow fragment of DNA polymerase. Proc. Nat. Acad. Sci. U.S.A. 2017; 114:9605–9610. 10.1073/pnas.1704258114 PMC5594654 28827350

[16] Phan A.T. , Guéron M., Leroy J.L. The solution structure and internal motions of a fragment of the cytidine-rich strand of the human telomere. J. Mol. Biol. 2000; 299:123–144. 10860727 10.1006/jmbi.2000.3613

[17] Garavís M. , Escaja N., Gabelica V., Villasante A., González C. Centromeric alpha-satellite DNA adopts dimeric i-motif structures capped by at Hoogsteen base pairs. Chem. Eur. J. 2015; 21:9816–9824. 26013031 10.1002/chem.201500448

[18] Zeraati M. , Langley D.B., Schofield P., Moye A.L., Rouet R., Hughes W.E., Bryan T.M., Dinger M.E., Christ D. I-motif DNA structures are formed in the nuclei of human cells. Nat. Chem. 2018; 10:631–637. 29686376 10.1038/s41557-018-0046-3

[19] Dzatko S. , Krafcikova M., Hänsel-Hertsch R., Fessl T., Fiala R., Loja T., Krafcik D., Mergny J.-L.L., Foldynova-Trantirkova S., Trantirek L. Evaluation of the stability of DNA i-motifs in the nuclei of living mammalian cells. Angew. Chem. 2018; 130:2165–2169. 10.1002/anie.201712284 PMC5820743 29266664

[20] Xu W. , Chan K.M., Kool E.T. Fluorescent nucleobases as tools for studying DNA and RNA. Nature Chem. 2017; 9:1043–1055. 29064490 10.1038/nchem.2859 PMC5819341

[21] Lin K.-Y. , Jones R.J., Matteucci M. Tricyclic 2’-deoxycytidine analogs: syntheses and incorporation into oligodeoxynucleotides which have enhanced binding to complementary RNA. J. Am. Chem. Soc. 1995; 117:3873–3874.

[22] Wilhelmsson L.M. , Holmén A., Lincoln P., Nielsen P.E., Nordén B. A highly fluorescent DNA base analogue that forms Watson−crick base pairs with guanine. J. Am. Chem. Soc. 2001; 123:2434–2435. 11456897 10.1021/ja0025797

[23] Reilly S.M. , Lyons D.F., Wingate S.E., Wright R.T., Correia J.J., Jameson D.M., Wadkins R.M. Folding and hydrodynamics of a DNA i-motif from the c-MYC promoter determined by fluorescent cytidine analogs. Biophys. J. 2014; 107:1703–1711. 25296324 10.1016/j.bpj.2014.08.014 PMC4190599

[24] Bielecka P. , Juskowiak B. Fluorescent sensor for pH monitoring based on an i-motif - switching aptamer containing a tricyclic cytosine analogue (tC). Molecules. 2015; 20:18511–18525. 26473815 10.3390/molecules201018511 PMC6332284

[25] Tsvetkov V.B. , Zatsepin T.S., Belyaev E.S., Kostyukevich Y.I., Shpakovski G.V., Podgorsky V.V., Pozmogova G.E., Varizhuk A.M., Aralov A.V. I-clamp phenoxazine for the fine tuning of DNA i-motif stability. Nucleic Acids Res. 2018; 46:2751–2764. 29474573 10.1093/nar/gky121 PMC5888743

[26] Mata G. , Luedtke N.W. Fluorescent probe for proton-coupled DNA folding revealing slow exchange of *i* -motif and duplex structures. J. Am. Chem. Soc. 2015; 137:699–707. 25423623 10.1021/ja508741u

[27] Bielecka P. , Dembska A., Juskowiak B. Monitoring of pH using an i-motif-forming sequence containing a fluorescent cytosine analogue, tC. Molecules. 2019; 24:952. 30857134 10.3390/molecules24050952 PMC6429216

[28] Dembska A. , Bielecka P., Juskowiak B. pH-sensing fluorescence oligonucleotide probes based on an i-motif scaffold: a review. Anal. Methods. 2017; 9:6092–6106.

[29] Preus S. , Kilså K., Wilhelmsson L.M., Albinsson B. Photophysical and structural properties of the fluorescent nucleobase analogues of the tricyclic cytosine (tC) family. Phys. Chem. Chem. Phys. 2010; 12:8881. 20532361 10.1039/c000625d

[30] Engman K.C. DNA adopts normal B-form upon incorporation of highly fluorescent DNA base analogue tC: NMR structure and UV-vis spectroscopy characterization. Nucleic Acids Res. 2004; 32:5087–5095. 15452275 10.1093/nar/gkh844 PMC521657

[31] Sandin P. , Börjesson K., Li H., Mårtensson J., Brown T., Wilhelmsson L.M., Albinsson B. Characterization and use of an unprecedentedly bright and structurally non-perturbing fluorescent DNA base analogue. Nucleic Acids Res. 2008; 36:157–167. 18003656 10.1093/nar/gkm1006 PMC2248743

[32] Preus S. , Börjesson K., Kilså K., Albinsson B., Wilhelmsson L.M. Characterization of nucleobase analogue FRET acceptor tC _nitro_. J. Phys. Chem. B. 2010; 114:1050–1056. 20039634 10.1021/jp909471b

[33] Baladi T. , Nilsson J.R., Gallud A., Celauro E., Gasse C., Levi-Acobas F., Sarac I., Hollenstein M.R., Dahlén A., Esbjörner E.K. et al. Stealth fluorescence labeling for live microscopy imaging of mRNA delivery. J. Am. Chem. Soc. 2021; 143:5413–5424. 33797236 10.1021/jacs.1c00014 PMC8154517

[34] Escaja N. , Pedroso E., Rico M., González C., Gonzàlez C., Escaja N., Pedroso E., Rico M., González C. Dimeric solution structure of two cyclic octamers: four-stranded DNA structures stabilized by A:T:A:T and G:C:G:C tetrads. J. Am. Chem. Soc. 2000; 122:12732–12742.

[35] Escaja N. , Gelpí J.L.J.L., Orozco M., Rico M., Pedroso E., González C. Four-stranded DNA structure stabilized by a novel G:C:A:T tetrad. J. Am. Chem. Soc. 2003; 125:5654–5662. 12733903 10.1021/ja0344157

[36] Escaja N. , Gómez-Pinto I., Pedroso E., González C. Four-stranded DNA structures can Be stabilized by two different types of Minor groove G:C:G:C tetrads. J. Am. Chem. Soc. 2007; 129:2004–2014. 17260988 10.1021/ja066172z

[37] Gallego J. , Chou S.H., Reid B.R. Centromeric pyrimidine strands fold into an intercalated motif by forming a double hairpin with a novel T:G:G:T tetrad: solution structure of the d(TCCCGTTTCCA) dimer. J. Mol. Biol. 1997; 273:840–856. 9367776 10.1006/jmbi.1997.1361

[38] Viladoms J. , Escaja N., Pedroso E., González C. Self-association of cyclic oligonucleotides through G:T:G:T minor groove tetrads. Bioorg. Med. Chem. 2010; 18:4067–4073. 20452223 10.1016/j.bmc.2010.04.018

[39] Escaja N. , Viladoms J., Garavís M., Villasante A., Pedroso E., González C. A minimal i-motif stabilized by minor groove G:T:G:T tetrads. Nucleic Acids Res. 2012; 40:11737–11747. 23042679 10.1093/nar/gks911 PMC3526289

[40] Escaja N. , Mir B., Garavís M., González C. Non-G base tetrads. Molecules. 2022; 27:5287. 36014524 10.3390/molecules27165287 PMC9414646

[41] Leonard G.A. , Zhang S., Peterson M.R., Harrop S.J., Helliwell J.R., Cruse W.B., Langlois d’Estaintot B., Kennard O., Brown T., Hunter W.N. Self-association of a DNA loop creates a quadruplex: crystal structure of d(GCATGCT) at 1.8 å resolution. Structure. 1995; 3:335–340. 7613864 10.1016/s0969-2126(01)00165-4

[42] Viladoms J. , Escaja N., Frieden M., Gómez-Pinto I., Pedroso E., González C. Self-association of short DNA loops through minor groove C:G:G:C tetrads. Nucleic Acids Res. 2009; 37:3264–3275. 19321501 10.1093/nar/gkp191 PMC2691830

[43] Escaja N. , Gómez-Pinto I., Viladoms J., Pedroso E., González C. The effect of loop residues in four-stranded dimeric structures stabilized by minor groove tetrads. Org. Biomol. Chem. 2013; 11:4804–4810. 23764570 10.1039/c3ob40741a

[44] Goddard D.T. , Kneller G. SPARKY. 2000; 3rd edn San Francisco University of California.

[45] Serrano-Chacón I. , Mir B., Escaja N., González C. Structure of i-motif/duplex junctions at neutral pH. J. Am. Chem. Soc. 2021; 143:12919–12923. 34370473 10.1021/jacs.1c04679 PMC8397320

[46] Bayly C.I. , Cieplak P., Cornell W., Kollman P.A. A well-behaved electrostatic potential based method using charge restraints for deriving atomic charges: the RESP model. J. Phys. Chem. 1993; 97:10269–10280.

[47] Cornell W.D. , Cieplak P., Bayly C.I., Gould I.R., Merz K., Ferguson D.M., Spellmeyer D.C., Fox T., Caldwell J.W., Kollman P.A. A 2nd generation force field for the simulation of proteins, nucleic acids and organic molecules. J. Am. Chem. Soc. 1995; 117:5179–5197.

[48] Ivani I. , Dans P.D.P.D., Noy A., Pérez A., Faustino I., Hospital A., Walther J., Andrio P., Goñi R., Balaceanu A. et al. Parmbsc1: a refined force field for DNA simulations. Nat. Methods. 2015; 13:55–58. 26569599 10.1038/nmeth.3658 PMC4700514

[49] Wang J. , Wolf R.M., Caldwell J.W., Kollman P.A., Case D.A. Development and testing of a general amber force field. J. Comput. Chem. 2004; 25:1157–1174. 15116359 10.1002/jcc.20035

[50] Güntert P. Automated NMR structure calculation with CYANA. Methods Mol. Biol. 2004; 278:353–378. 15318003 10.1385/1-59259-809-9:353

[51] Case D.A. , Pearlman D.A., Caldwell J.W. III, T. E.C., Wang J., Ross W.S., Simmerling C.L., Darden T.A., Merz K.M., Stanton R.V. et al. 2018; AMBER 18.

[52] Jorgensen W.L. Revised TIPS for simulations of liquid water and aqueous solutions. J. Chem. Phys. 1982; 77:4156.

[53] Koradi R. , Billeter M., Wüthrich K. MOLMOL: a program for display and analysis of macromolecular structures. J. Mol. Graphics. 1996; 14:29–32. 10.1016/0263-7855(96)00009-4 8744573

[54] Lu X.J. , Olson W.K. 3DNA: a versatile, integrated software system for the analysis, rebuilding and visualization of three-dimensional nucleic-acid structures. Nat. Protoc. 2008; 3:1213–1227. 18600227 10.1038/nprot.2008.104 PMC3065354

[55] Gaussian 16, Revision B.01 Frisch M.J. , Trucks G.W., Schlegel H.B., Scuseria G.E., Robb M.A., Cheeseman J.R., Scalmani G., Barone V., Petersson G.A., Nakatsuji H. 2016; Gaussian, Inc., Wallingford CT Gausssian 16.

[56] Becke A.D. Density‐functional thermochemistry. III. The role of exact exchange. J. Chem. Phys. 1993; 98:5648–5652.

[57] Scalmani G. , Frisch M.J. Continuous surface charge polarizable continuum models of solvation. I. General formalism. J. Chem. Phys. 2010; 132:114110. 20331284 10.1063/1.3359469

[58] Grimme S. , Ehrlich S., Goerigk L. Effect of the damping function in dispersion corrected density functional theory. J. Comput. Chem. 2011; 32:1456–1465. 21370243 10.1002/jcc.21759

[59] Boys S.F. , Bernardi F. The calculation of small molecular interactions by the differences of separate total energies. Some procedures with reduced errors. Mol. Phys. 1970; 19:553–566.

[60] Karimi A. , Wang K., Basran K., Copp W., Luedtke N.W. A bright and ionizable cytosine mimic for i-motif structures. Bioconjugate Chem. 2023; 34:972–976. 10.1021/acs.bioconjchem.3c00055 37196003

[61] Karimi A. , Börner R., Mata G., Luedtke N.W. A highly fluorescent nucleobase molecular rotor. J. Am. Chem. Soc. 2020; 142:14422–14426. 32786749 10.1021/jacs.0c05180

[62] Watari Y. , Nakatani K., Matsuo K., Waku T., Kobori A. Wash-free FISH of bacterial ribosomal RNAs by benzo[a]pyrene-modified oligonucleotides. Results Chem. 2024; 7:101214.

[63] Zhou P. , Han K. Unraveling the detailed mechanism of excited-State proton transfer. Acc. Chem. Res. 2018; 51:1681–1690. 29906102 10.1021/acs.accounts.8b00172

[64] Nesterova I.V. , Nesterov E.E. Rational design of highly responsive pH sensors based on DNA i-Motif. J. Am. Chem. Soc. 2014; 136:8843–8846. 24901653 10.1021/ja501859w

[65] Petrunina N.A. , Shtork A.S., Lukina M.M., Tsvetkov V.B., Khodarovich Y.M., Feofanov A.V., Moysenovich A.M., Maksimov E.G., Shipunova V.O., Zatsepin T.S. et al. Ratiometric i-motif-based sensor for precise long-term monitoring of pH micro alterations in the nucleoplasm and interchromatin granules. ACS Sens. 2023; 8:619–629. 36662613 10.1021/acssensors.2c01813

[66] Dong Y. , Yang Z., Liu D. DNA nanotechnology based on i-motif structures. Acc. Chem. Res. 2014; 47:1853–1860. 24845472 10.1021/ar500073a

[67] Yatsunyk L.A. , Mendoza O., Mergny J.-L. “Nano-oddities”: unusual nucleic acid assemblies for DNA-based nanostructures and nanodevices. Acc. Chem. Res. 2014; 47:1836–1844. 24871086 10.1021/ar500063x

[68] Alba J.J. , Sadurní A., Gargallo R. Nucleic acid *i-* motif structures in analytical chemistry. Crit. Rev. Anal. Chem. 2016; 46:443–454. 26939549 10.1080/10408347.2016.1143347

[69] Boissieras J. , Bonnet H., Susanto M.F., Gomez D., Granzhan A., Defrancq E., Dejeu J. iMab antibody binds single-stranded cytosine-rich sequences and unfolds DNA i-motifs biophysics. 2023; bioRxiv doi:21 November 2023, preprint: not peer reviewed 10.1101/2023.11.21.568054. PMC11317162 38908025

[70] Zanin I. , Ruggiero E., Nicoletto G., Lago S., Maurizio I., Gallina I., Richter S.N. Genome-wide mapping of i-motifs reveals their association with transcription regulation in live human cells. Nucleic Acids Res. 2023; 51:8309–8321. 37528048 10.1093/nar/gkad626 PMC10484731

[71] Trantirek L. , Viskova P., Istvankova E., Rynes J., Dzatko S., Loja T., Zivkovic M.L., Rigo R., El-Khoury R., Serrano-Chacón I. et al. DNA i-motif levels are overwhelmingly depleted in living human cells: insights from in-cell NMR. 2024; bioRxiv doi:02 October 2023, preprint: not peer reviewed 10.1101/2023.10.01.558881. PMC10914786 38443388

