# Supplementary material for: Site-specific incorporation of a fluorescent nucleobase analog enhances i-motif stability and allows monitoring of i-motif folding inside cells
Source: Nucleic Acids Res. 2024 Feb 16;52(6):3375–89. doi: 10.1093/nar/gkae106 (PMC11014255; doi:10.1093/nar/gkae106)
Supplement: gkae106_Supplemental_File [file gkae106_supplemental_file.pdf]

SUPPLEMENTARY DATA

Site-specific incorporation of a fluorescent nucleobase analog enhances i-motif stability and allows monitoring its folding inside cells

Bartomeu Mir,[1,2] Israel Serrano-Chacón,[1,3] Pedro Medina,[3,4] Veronica Macaluso,[3] Montserrat Terrazas,[&,‡] Albert Gandioso,[3] Miguel Garavís,[1,§] Modesto Orozco,[3,4,*] Núria Escaja,[2,§,*] and Carlos González[1,§*].

[1] Instituto de Química Física ''Blas Cabrera''. CSIC. Serrano 119. 28006 Madrid. Spain

[2] Inorganic and Organic Chemistry Department. Organic Chemistry Section. and IBUB. University of Barcelona. Martí i Franquès 1-11. 08028 Barcelona. Spain

[3] Institute for Research in Biomedicine (IRB Barcelona). The Barcelona Institute of Science and Technology (BIST). 08028 Barcelona. Spain

[4] Departament de Bioquímica i Biomedicina. Facultat de Biologia. Universitat de Barcelona. 08028 Barcelona. Spain

§ BIOESTRAN associated unit UB-CSIC

Supplementary Figures

Figure S1. NMR, CD spectra and UV melting curves of **NN4_tC^{O}2** in K$^+$ buffer. S-2
Figure S2. Fluorescence excitation and emission spectra of **NN4_tC^{O}2** and **NN4_tC^{O}6.** S-3
Figure S3. Fluorescence excitation spectra for i-motif and duplex forms of **NN4_tC^{O}2** at pH 7. S-4
Figure S4. Exchangeable protons region of NOESY spectra of **NN4_tC^{o}2** at pH 7 (T = 5 ^{o}C). S-5
Figure S5. Non-exchangeable protons regions (Ar-H1'/H2'/H2") of NOESY of **NN4_tC^{o}2** at pH 7 (T = 20 ^{o}C). S-6
Figure S6. Non-exchangeable protons regions (Ar-H3'/H4'/H5'/H5") of NOESY of **NN4_tC^{o}2** at pH 7 (T = 20 ^{o}C). S-7
Figure S7. Scheme of the most relevant NOE contacts found for **NN4_tC^{o}2** at pH 7. S-8
Figure S8. Three different views of the superposition of 10 refined structures of **NN4_tC^{O}2.** S-9
Figure S9. Comparison of the structures of **NN4_tC^{O}2** and **NN4.** S-10
Figure S10. Exchangeable protons regions of NOESY spectra of **NN4_tC^{o}2** at pH 6 (T = 5 ^{o}C). S-11
Figure S11. Exchangeable protons regions of NOESY spectra of **NN4_tC^{o}2** at pH 5 (T = 5 ^{o}C). S-11
Figure S12. Exchangeable protons regions of NOESY spectra of **NN4_tC^{o}2** at pH 4 (T = 5 ^{o}C). S-12
Figure S13. Non-exchangeable protons regions of NOESY spectra of **NN4_tC^{o}2** at pH 4 (T = 5 ^{o}C). S-13
Figure S14. Non-exchangeable protons regions of NOESY spectra of **NN4_tC^{o}6** at pH 7 (T = 5 ^{o}C). S-14
Figure S15. Exchangeable protons regions of NOESY spectra of **NN4_tC^{o}6** at pH 7 (T = 5 ^{o}C). S-15
Figure S16. Exchangeable protons regions of NOESY spectra of **NN4_tC^{o}6** at pH 5 (T = 5 ^{o}C). S-16
Figure S17. QM calculations**.** S-17
Figure S18. Three-dimensional representation of the CD-monitored pH-titration experiments. S-18
Figure S19. CD-monitored pH titration curves at 295 nm. S-18
Figure S20. Summary of the different equilibria observed in **NN4** and its modified analogs. S-19
Figure S21. NMR and fluorescence excitation and emission spectra of **controlNN4_tC^{O}2.** S-20
Figure S22. pHrodoTM green fluorescence calibration in different cells. S-21
Figure S23. Variation of pHrodoTM green fluorescence versus pH in fixed cells. S-22
Figure S24. Fluorescence emission of transfected HeLa cells at acidic pH (6.5). S-23
Figure S25. Fluorescence emission of transfected HeLa cells at physiological pH (7.4). S-24
Figure S26. Fluorescence emission of transfected HeLa cells at alkaline pH (8.5). S-25
Figure S27. MALDI-TOF spectra of the synthesized oligonucleotides. S-26

Supplementary Tables

Table S1. Chemical shift lists of **NN4_tC^{O}2** (pH 7, T=5 ºC). S-27

Table S2. Chemical shift lists of non-exchangeable protons of **NN_4tC^{O}2** (pH 7, T=20 ºC). S-28

Table S*3*. Experimental constraints and calculation statistics of **NN4_tC^{O}2** at pH 7. S-29

T**able S4.** Average dihedral angles and order parameters of the neutral structure of **NN4_tC^{O}2.** S-29

Table S5. Pseudorotation angles and amplitude values of **NN4_tC^{O}2.** S-30

Table S*6*. Molar extinction coefficients of **NN4** sequences. S-30

Table S*7*. Calculated HOMO-LUMO energies values (eV) in water. S-30

Supplementary Figures

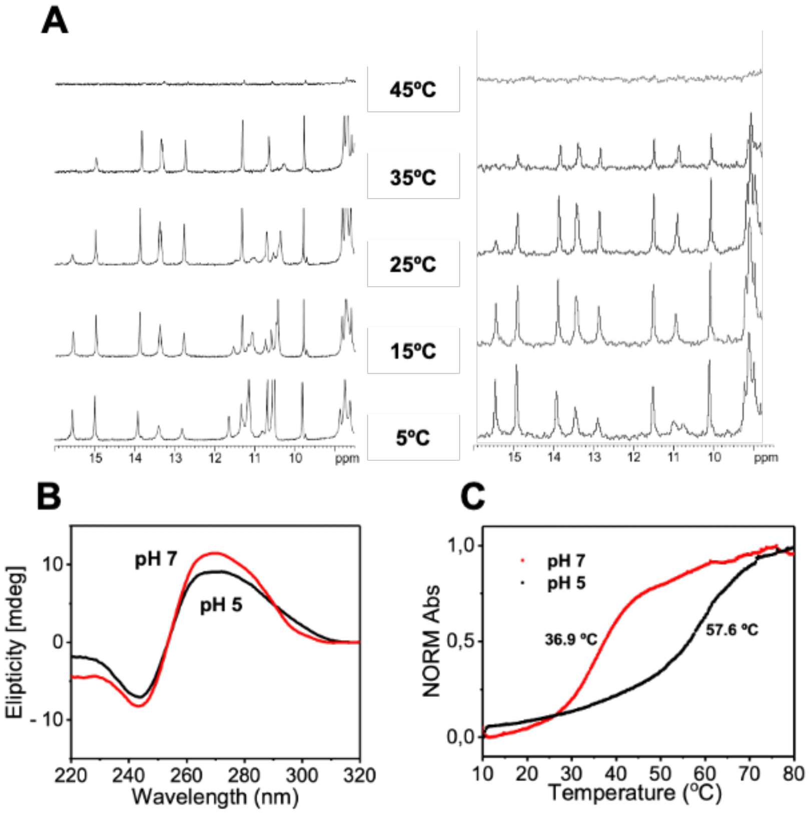

Figure S1. A) ^{1}H-NMR spectra at different temperatures of **NN4_tC^{O}2,** 10 mM sodium phosphate buffer, [oligonucleotide] = 1 mM (left) and 10 mM potassium phosphate buffer, 100 mM KCl and [oligonucleotide] = 0.2 mM (right). B) CD spectra of **NN4_tC^{O}2** at pH 7 (red) and pH 5 (black), 10 mM potassium phosphate buffer, 100 mM KCl and [oligonucleotide] = 0.2 mM. C) UV melting curves of **NN4_tC^{O}2** pH 7 (red, Tm = 36.9 ºC) and pH 5 (black, Tm = 57.6 ºC), 10 mM potassium? phosphate buffer, 100 mM KCl and [oligonucleotide] = 0.2 mM

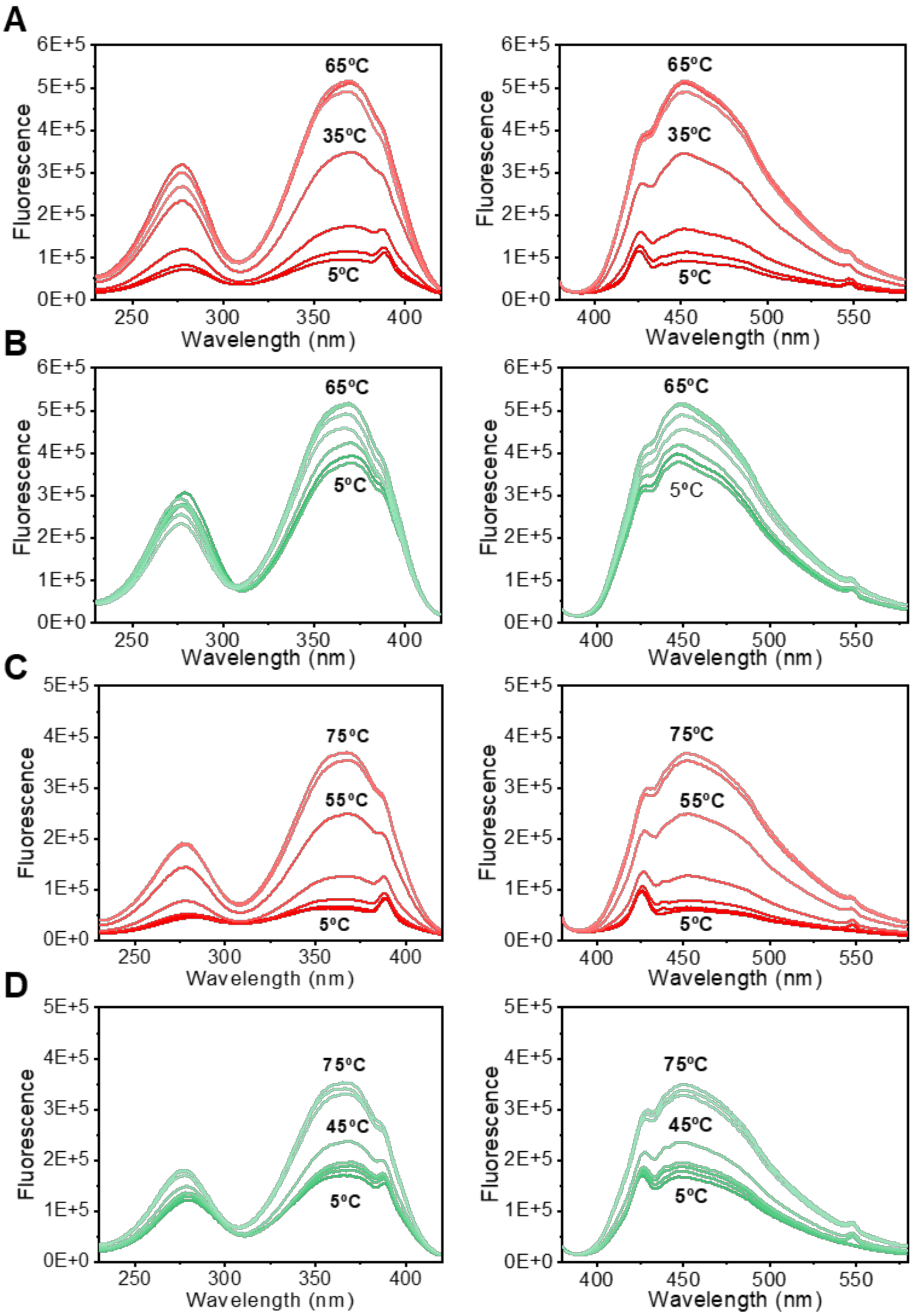

***Figure S2*.-** Fluorescence excitation (left) and emission spectra (right) recorded at different temperature for **NN4_tC^{O}2** (A,C) and **NN4_tC^{O}6** (B,D) at pH 7 (A,B) and pH 5 (C,D). 25 mM sodium phosphate buffer, [oligonucleotide] = 0.2 µM.

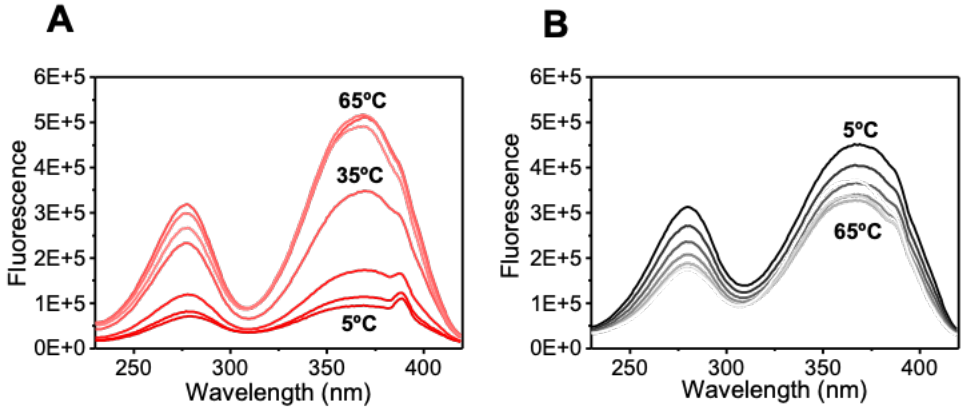

Figure S3.- Fluorescence excitation spectra recorded at different temperature for **NN4_tC^{O}2** (left) and **NN4_tC^{O}2** in presence of its complementary sequence (right) at pH 7. 25 mM sodium phosphate buffer, [oligonucleotide] = 0.2 µM.

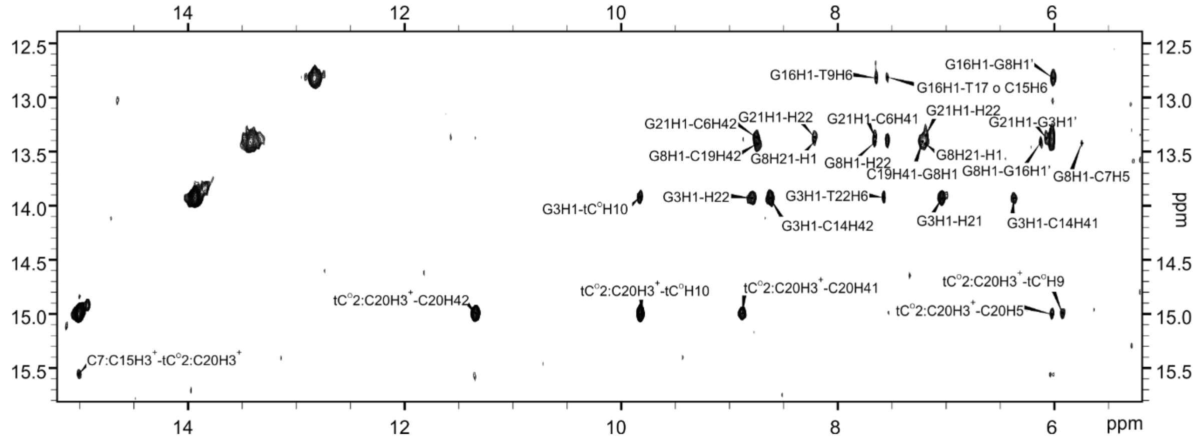

Figure S4.- Exchangeable protons region (down side of the diagonal) of NOESY (150 ms) of **NN4_tC°2** at pH 7, T= 5 °C. 10 mM sodium phosphate buffer (H_2O/D_2O 90:10), [oligonucleotide] = 1mM.

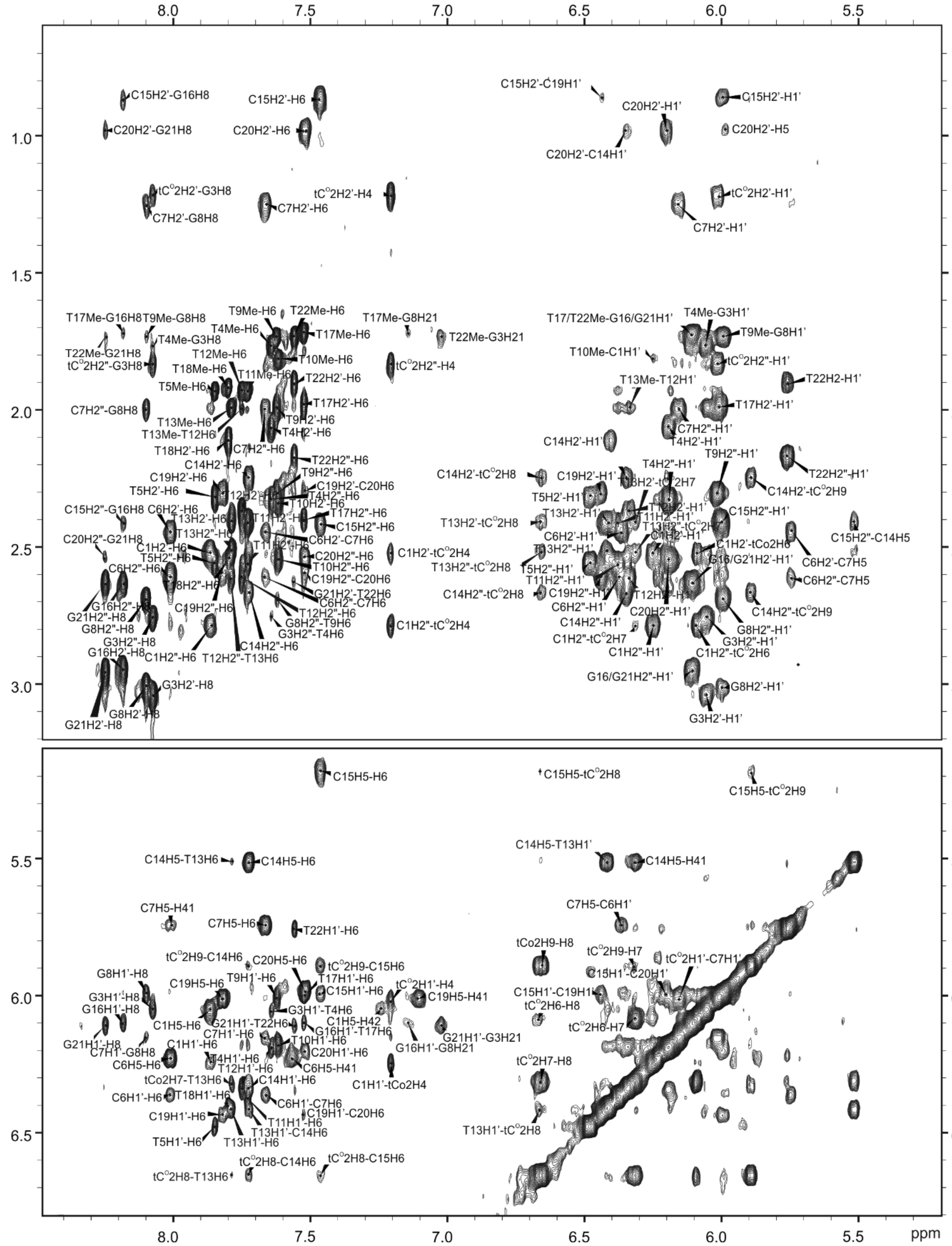

Figure S5.- Non-exchangeable protons regions of NOESY (250 ms) of **NN4_tC^{o}2** at pH 7, T= 20 ^{o}C. 10 mM sodium phosphate buffer (H_2O/D_2O 90:10), [oligonucleotide] = 1mM.

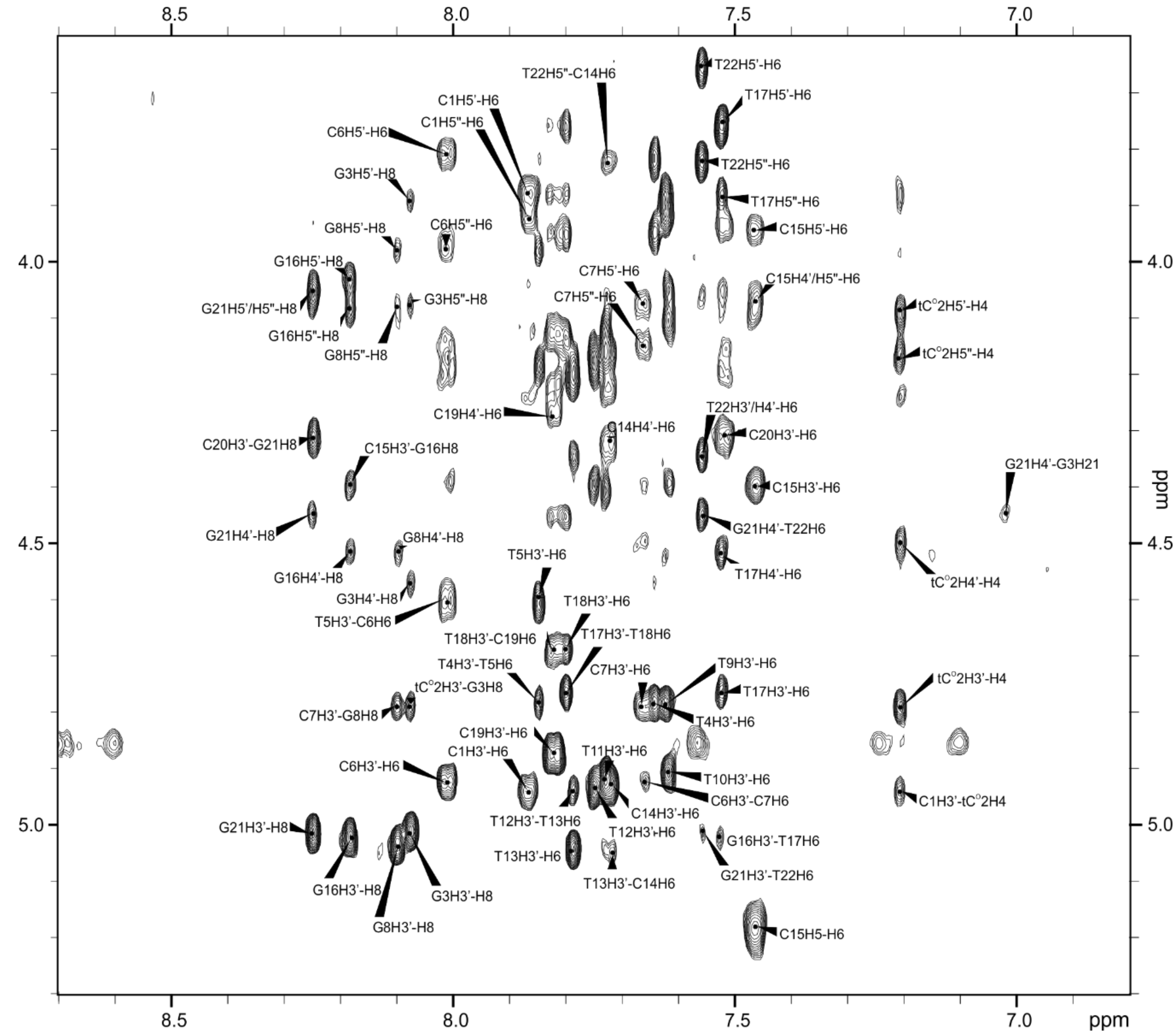

Figure S6.- Non-exchangeable protons Ar-H3’/H4’/H5’/H5” region of NOESY (250 ms) of **NN4_tC^{o}2** at pH 7, T= 20 ºC. 10 mM sodium phosphate buffer (H_2O/D_2O 90:10), [oligonucleotide] = 1mM.

Assignment of NN4_tC^{O}2 at neutral pH. NOEs involving tC^{O} were good starting points for the sequential assignment. H10 proton of tC^{O}2 shows cross-peaks with a couple of H2’/H2” protons of a cytosine involved in a G:C W-C base pair. Since in i-motifs, this kind of contacts are characteristic of 3’-3’ (back-to-back) intercalated cytosine residues, we assigned them to the cytosine base-paired to G3. Several cross-peaks confirm the stacking between these two residues: tC^{O}2H8-CH2’/H2”/H5, tC^{O}2H7-CH5 and tC^{O}2H9-CH2’/H2”/H3’. This cytosine and tC^{O}2 show cross-peaks with the same thymine residue (tC^{O}2H8-TH1’/H2’/H2”/H6, tC^{O}2H7-TH2’/H2”, and TH1’/H2’/H2”-CH5/H6), being those between the cytosine and the thymine typical sequential cross-peaks. All these contacts are consistent with the head-to-tail folding shown in Figure 1. Additionally, H4 proton of tC^{O}2 show cross-peaks with its own H2’/H2” protons, and the H2’/H2” protons of the previous cytosine in the sequence (C1). tC^{O}2H8 also exhibits some contacts with the 5’-5’ intercalated cytosine, which corresponds with that base paired to C7, that is C15 according to the proposed topology. Other sequential H6/H8-H1’/H2’/H2” NOESY cross-peaks between non-exchangeable protons were observed for the segments: C1-tC^{O}2-G3-T4, C6-C7-G8-T9, C15-G16-T17 and C19-C20-G21-T22 and some involving thymines of the central loop. Interestingly, cross-peaks H6/H8(n)-H3’(n-1) allows confirming all the sequence, including the central loop, except for the step C19-C20 (see ***Figure S5*** and ***Figure S6***). Some characteristic H1’-H1’ cross-peaks across the minor groove between intercalated residues were also observed confirming the stacking order: tC^{O}2-C7 and C19-C15-C20. Other additional cross-peaks were found in this region: C7H5-C6H1’, C14H5-T13H1’ and those corresponding to the tC^{O}2 aromatic system. Interestingly, H5 proton of C15 and C14 exhibit unusual upshifting, most pronounced in C15, probably due to the interaction across the major grove with the aromatic system of tC^{O}2. This unusual chemical shift near water signal prevents the observation of C15 H5-H41/H42 cross-peaks (see ***Figure S5***).

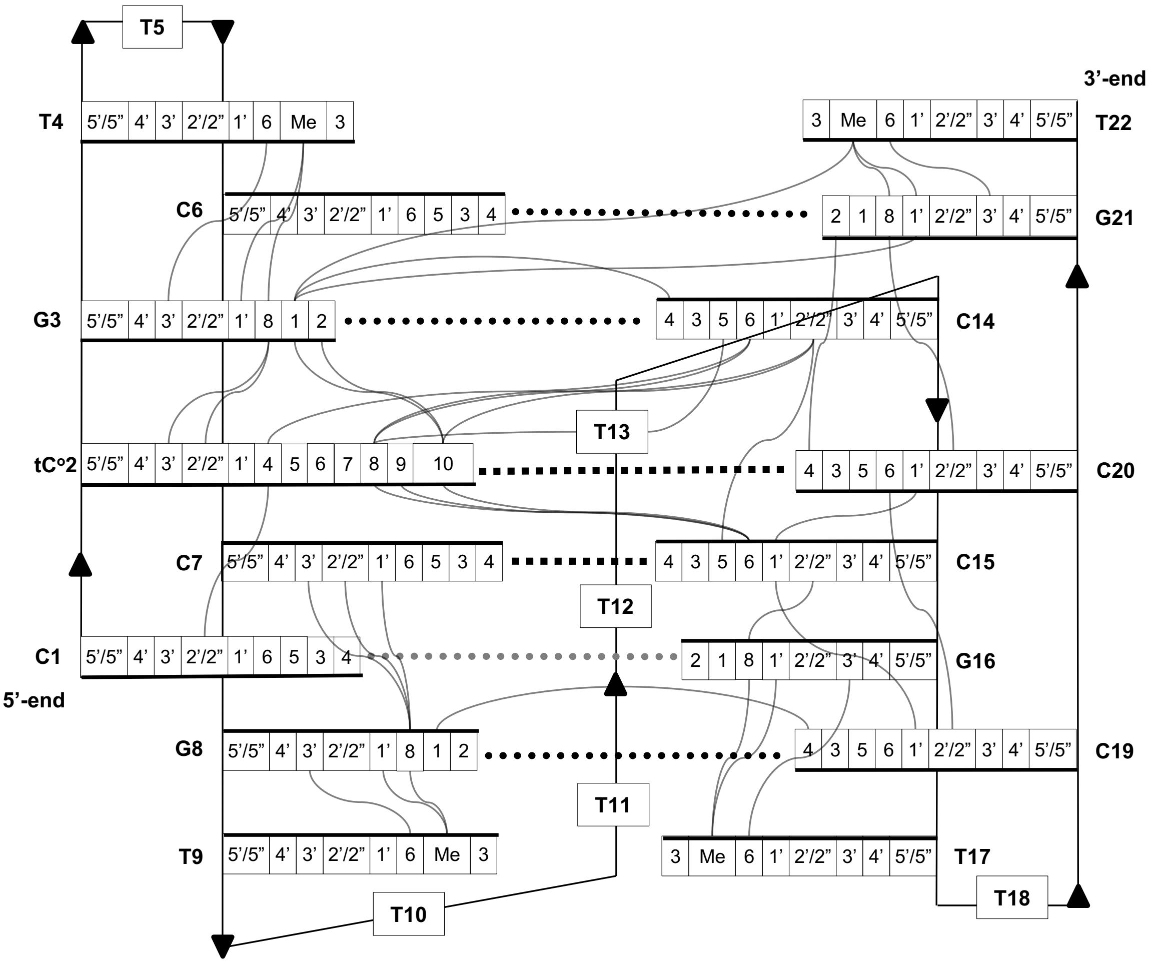

Figure S7.- Scheme of the most relevant NOE contacts found for **NN4_tC^{O}2** at pH 7. Round dotted lines indicate G:C base pairs and square dotted lines stand for C:C^{+} base pairs. C1:G16 base pair can be potentially formed but, as characteristics cross-peaks have not been observed, it is indicated in grey.

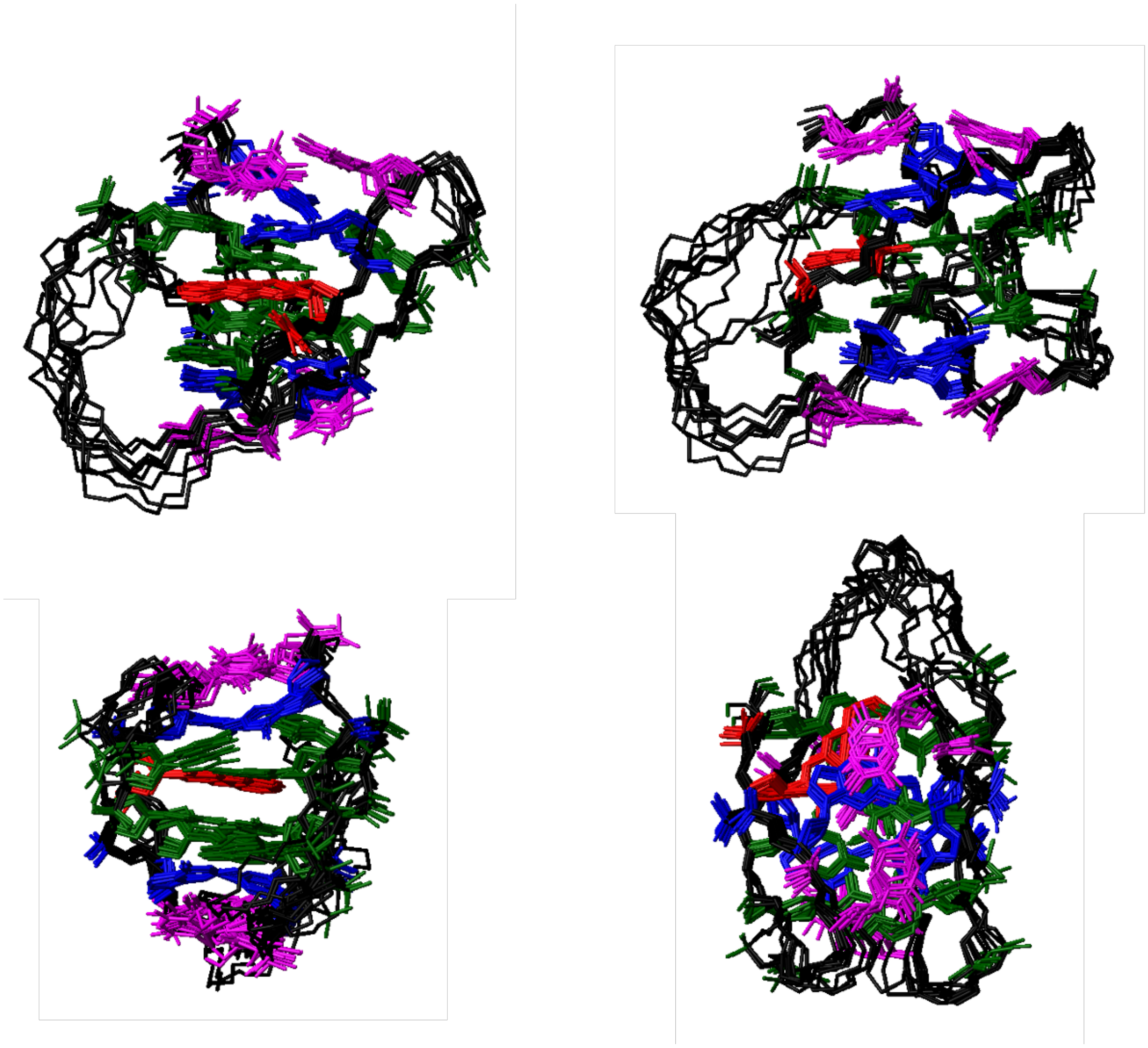

Figure S8.- Four different views of the superposition of 10 refined structures of **NN4_tC^{O}2.** Color code: tC^{O} in red, cytosines in green, guanines in blue, and well-defined thymines in magenta. Non-well-defined thymines (5,10,11,12,13,18) are not shown. Backbone is shown in black. (PDB: 8OFC)

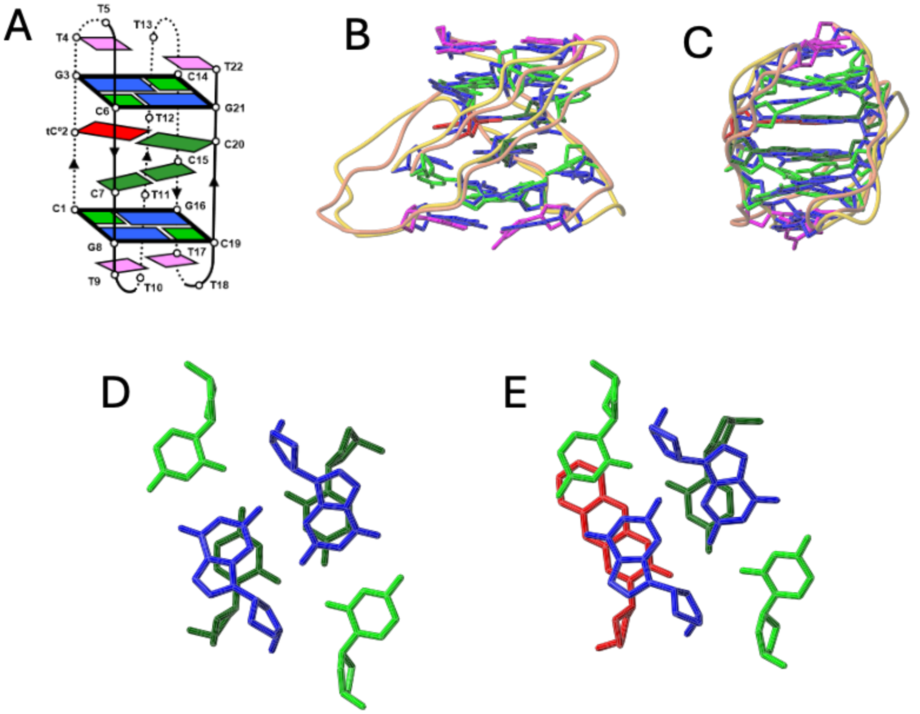

Figure S9.- Comparison of the neutral structures of **NN4_tC^{O}2** (PDB: 8OFC) and **NN4** (PDB: 8BV6). A) Scheme of **NN4_tC^{O}2**. B and C) Two views of the superposition of two representative structures **NN4_tC^{O}2** and **NN4**. Disordered thymines in the loops are not displayed. D, E) Details of the stacking the minor groove G:C:G:C tetrad with the C:C^{+} or tC^{O}:C^{+} base pair in **NN4** and **NN4_tC^{O}2**, respectively. Color code: tC^{O} in red, neutral cytosines in light green, hemiprotonated cytosines in dark green, guanines in blue, and well-defined thymines in magenta. Non-well-defined thymines (5,10,11,12,13,18) are not shown. Backbones are shown as ribbons in top figures.

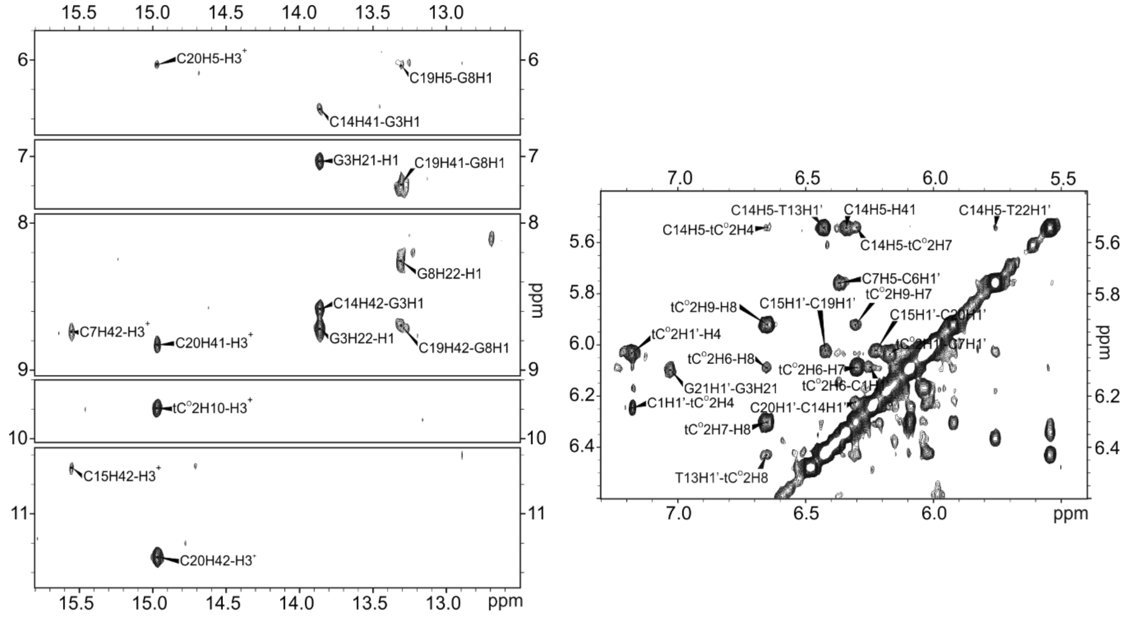

Figure S10.- Exchangeable and tC^{O} protons regions of NOESY (150 ms) of **NN4_tC^{o}2** at pH 6, T=5 ^{o}C. 10 mM sodium phosphate buffer (H_2O/D_2O 90:10), [oligonucleotide] = 0.43 mM.

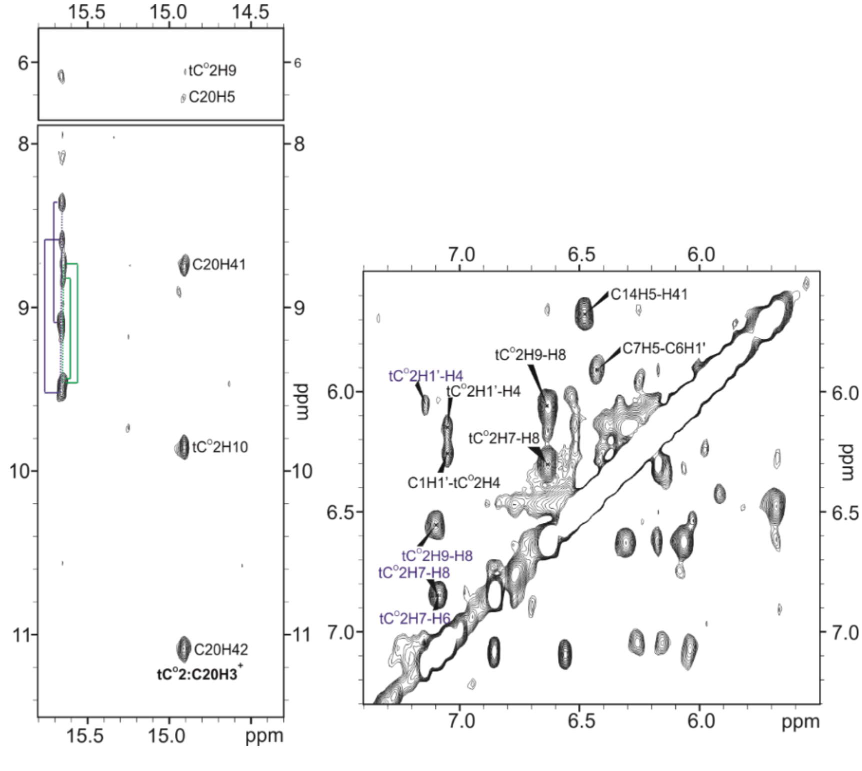

Figure S11.- Exchangeable and tC^{O} protons regions of NOESY (150 ms) of **NN4_tC^{o}2** at pH 5, T=5 ^{o}C. 10 mM sodium phosphate buffer (H_2O/D_2O 90:10), [oligonucleotide] = 0.43 mM. Up to 11 protonated imino-amino protons cross-peaks are found in the 15 ppm region. Formation of tC^{O}2:C20^{+} base pair corresponding to the neutral form is still clearly observed but at least two new C:C^{+} base pairs are also formed (blue and green lines). In the aromatic region, two set of tC^{O} protons (labelled in black (neutral form) and blue (acidic form)), respectively are clearly observed.

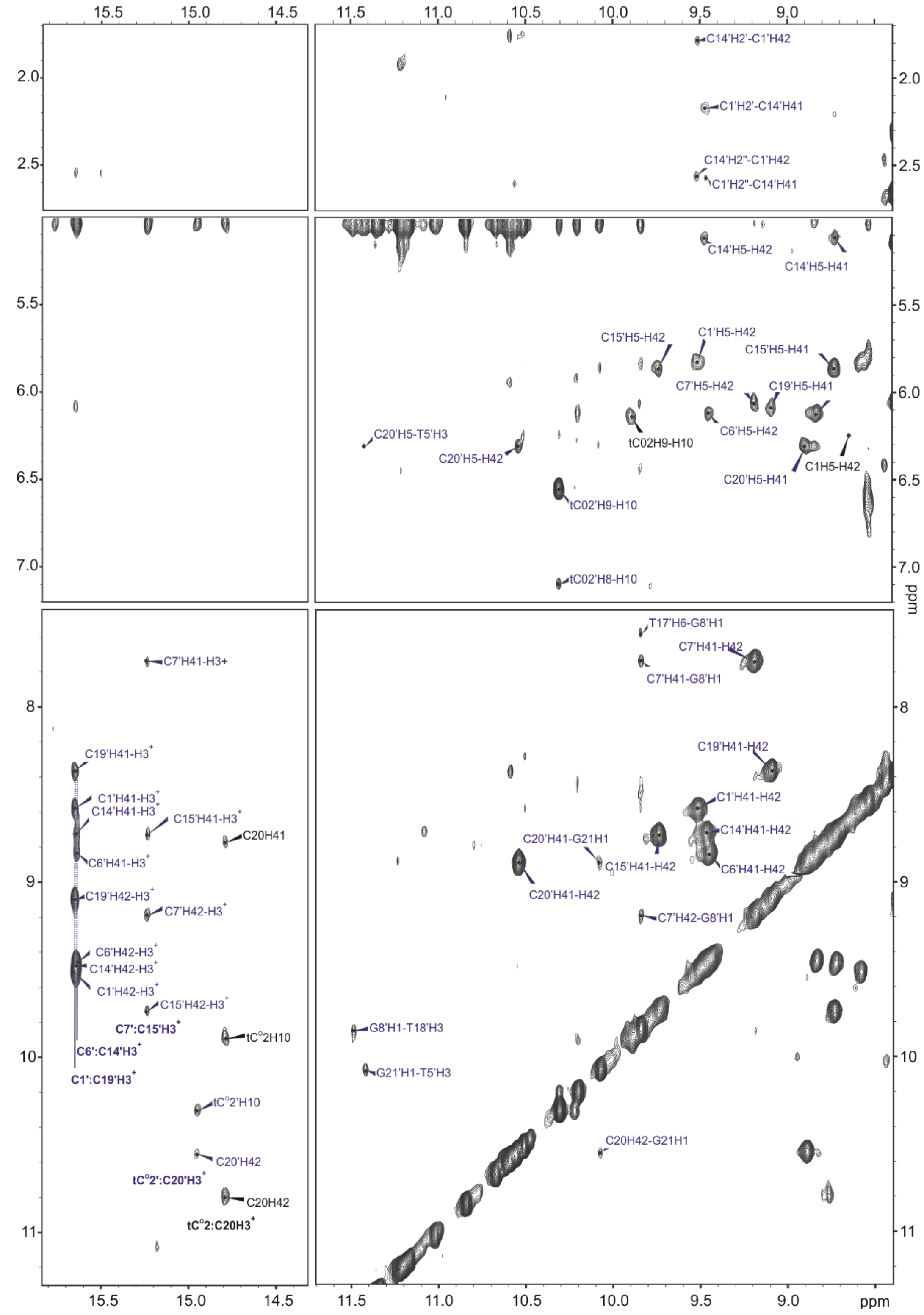

Figure S12.- Exchangeable protons regions of NOESY (150 ms) of **NN4_tC^{O}2** at pH 4, T=5 ^{o}C. 10 mM sodium phosphate buffer (H_2O/D_2O 90:10), [oligonucleotide] = 0.43 mM. Signals corresponding to the acidic form are shown in blue. Some signals corresponding to the neutral form are still observed (black labels).

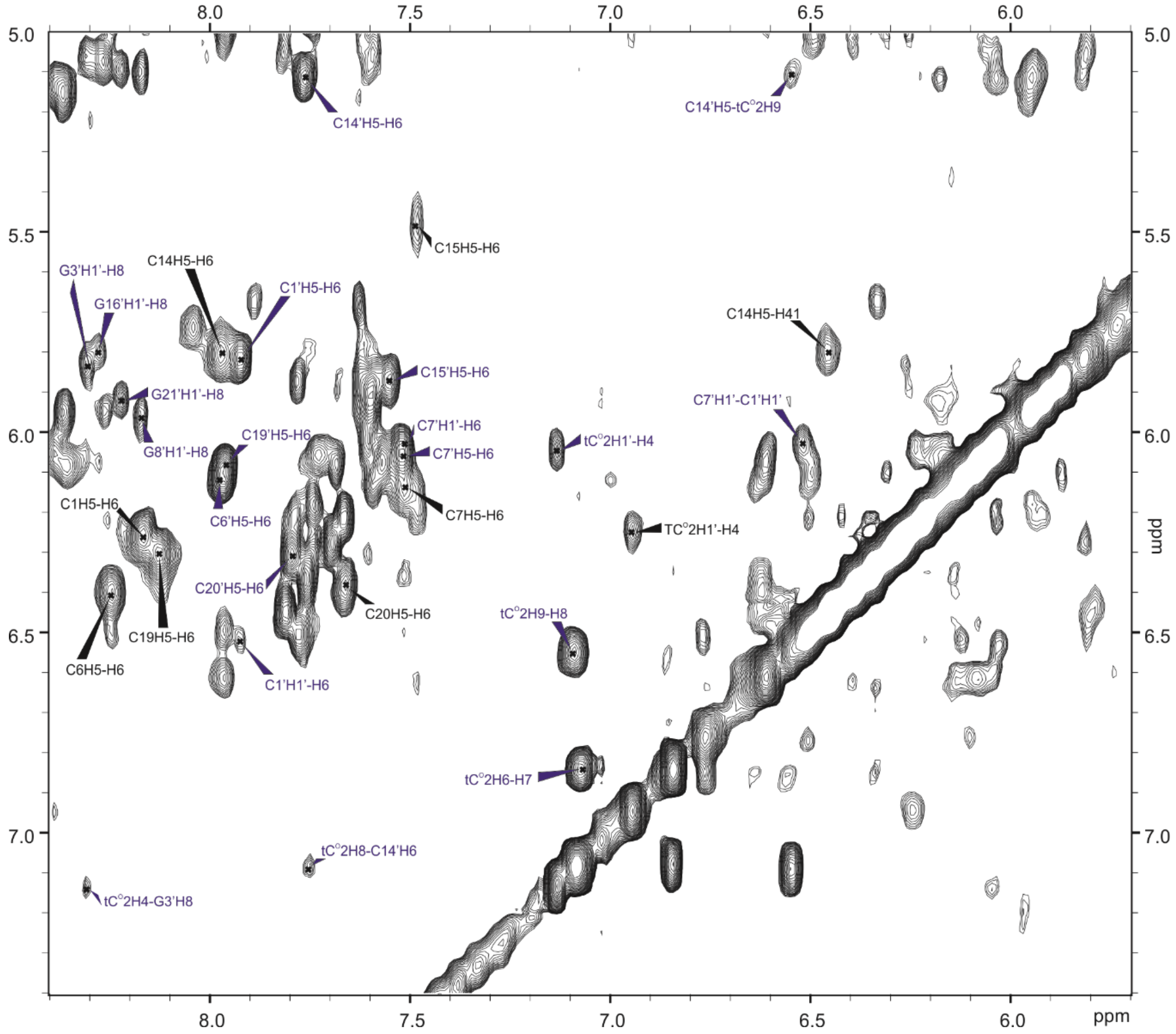

Figure S13.- Non-exchangeable protons regions of NOESY (150 ms) of **NN4_tC°2** at pH 4, T=5 °C. 10 mM sodium phosphate buffer (H_2O/D_2O 90:10), [oligonucleotide] = 0.43 mM. Signals corresponding to the acidic form are shown in blue. Some signals corresponding to the neutral form are still observed (black labels).

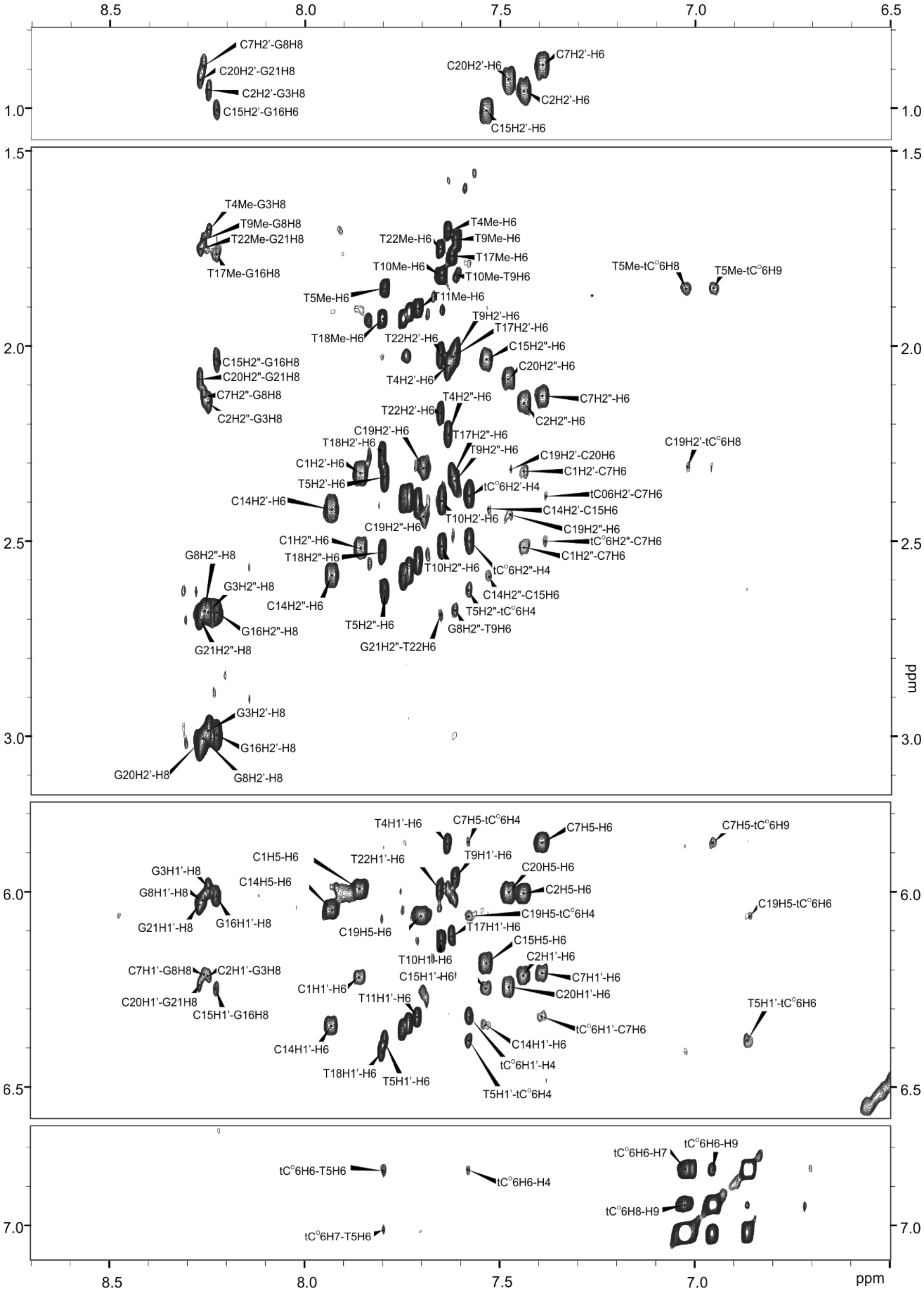

Figure S14.- Non-exchangeable protons regions of NOESY (250 ms) of **NN4_tC^{O}6** at pH 7, T= 5 ^{o}C. 10 mM sodium phosphate buffer (H_2O/D_2O 90:10), [oligonucleotide] = 1 mM.

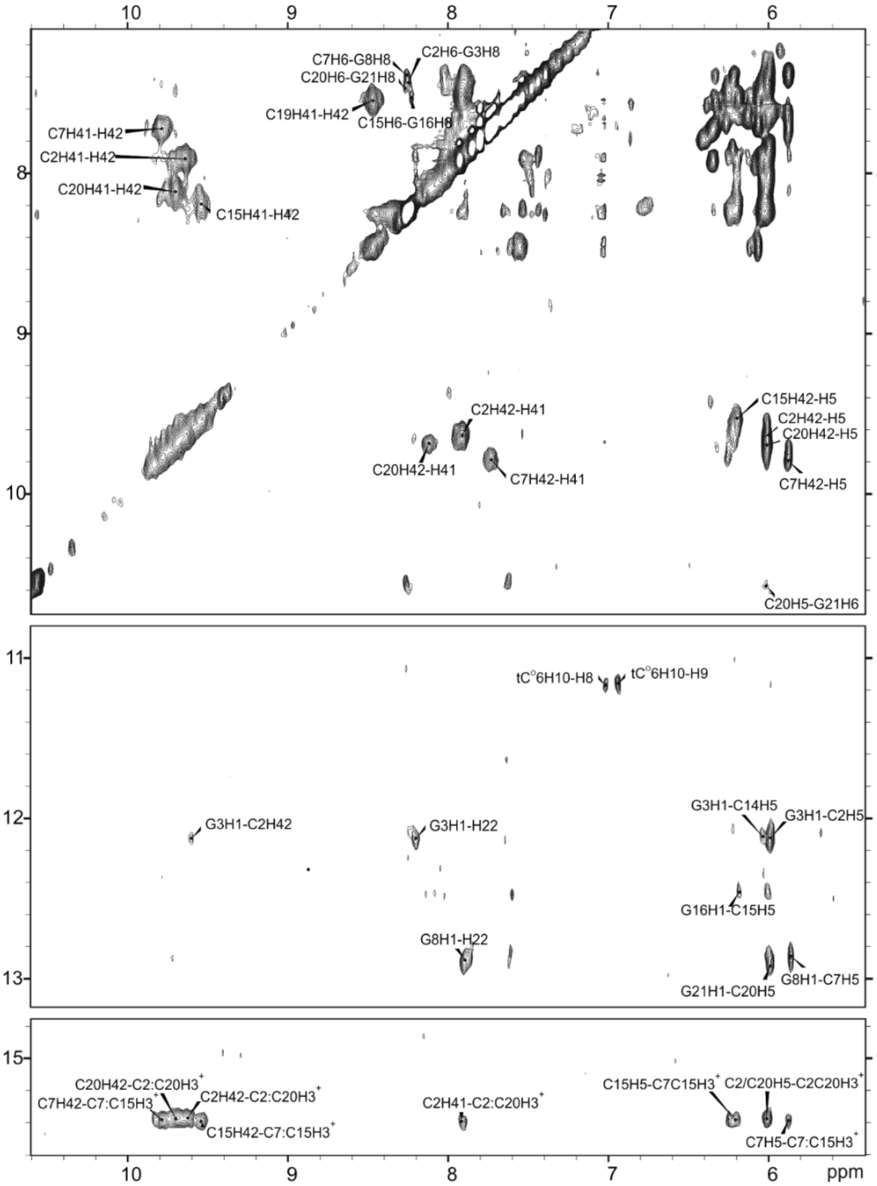

Figure S15.- Exchangeable and tC^O protons regions of NOESY (150 ms) of **NN4_tC^{O}6** at pH 7, T=5 ^{o}C. 10 mM sodium phosphate buffer (H_2O/D_2O 90:10), [oligonucleotide] = 1 mM.

Assignment of NN4_tC^{O}6 at neutral pH. Although the quality of these spectra is not enough to confirm the topology unambiguously, cross-peaks involving thymine connecting loop residues pointed to a head-to-tail folding with the thymine loop located near the major groove, as observed for other sequences of the family. Starting from tC^{O}6, considering stacking connections for the fragment T5-tC^{O}6-C7-G8-T9 and assuming a head-to-tail topology, the rest of the assignment could be carried out. The four cytosines involved in hemiprotonated base pairs could be matched as C7:C15$^+$ (15.40 ppm) and C2:C20$^+$ (15.38 ppm). Accordingly, the fragment C14-C15-G16-T17 could be identified. The observed contacts between tC^{O}6 and a cytosine not involved in hemiprotonated base pairs could be assigned to C19H3'/H5-tC^{O}6H8/H9, and would correspond to major groove contacts between tetrads residues, previously observed in this type of structures. From C19, the fragment C19-C20-G21-T22 could be completed. The remaining fragment, C1-C2-G3-T4, was assigned based on the formation of base C2:C20$^+$ pair. Only some of the thymine residues of the connecting loop could be assigned tentatively. Imino signals of hemiprotonated base pairs are clearly detected but characteristic G:C imino-amino cross-peaks are poorly observed. Iminos signals of G3, G8, G16 could be assigned on the basis of stacking cross-peaks with C2, C7 and C15, respectively. Although no cross-peak between tC^{O}6H10 proton and a guanine imino proton was observed, the chemical shift of this H10 proton (11.17 ppm) indicates its involvement in hydrogen bond formation.

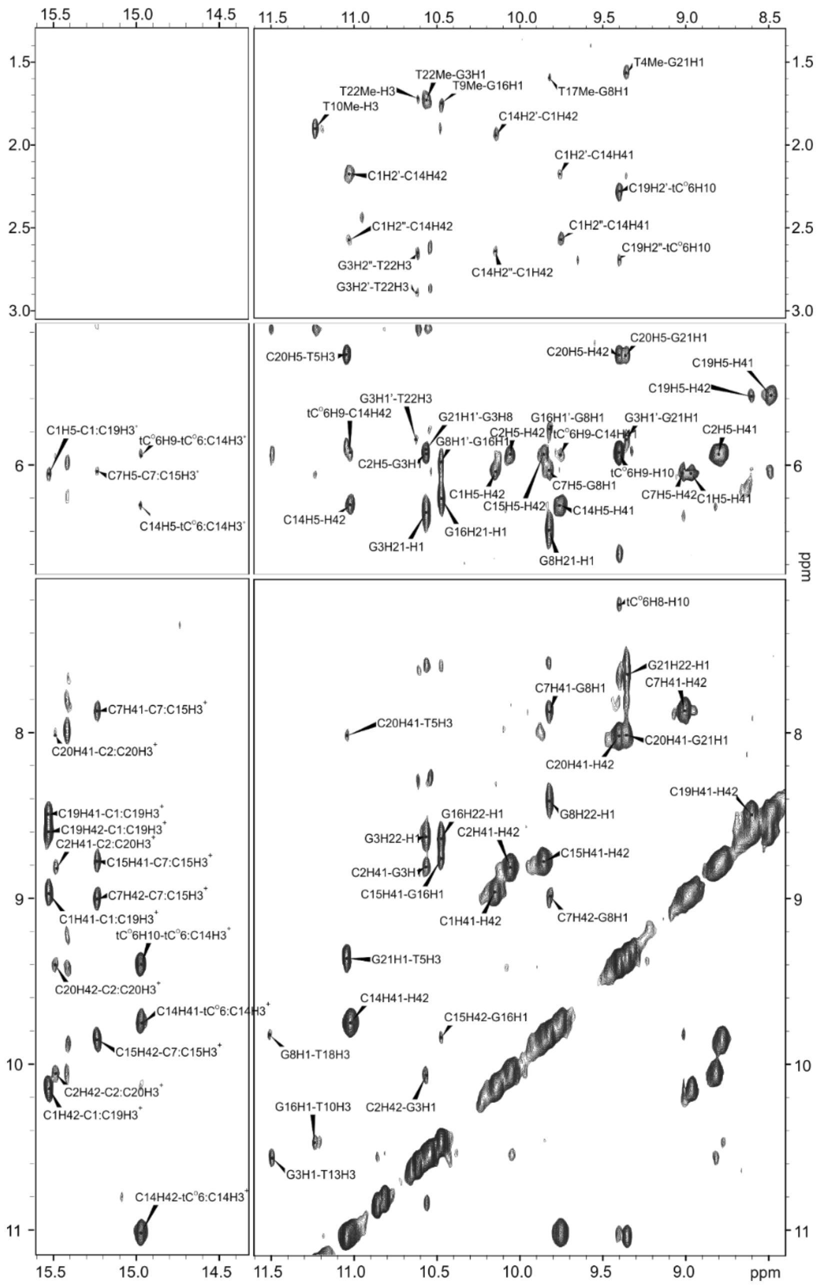

Figure S16.- Exchangeable and tC^{O} protons regions of NOESY (150 ms) of **NN4_tC^{O}6** at pH 5, T=5 ^{o}C. 10 mM sodium phosphate buffer (H_2O/D_2O 90:10), [oligonucleotide] = 1 mM.

Assignment of NN4_tC^{O}6 at acidic pH. At these conditions, tC^{O}6 residue is forming an hemiprotonated base pair that, according to a number of amino-H2'/H2" contacts across the major groove between 3'-3' intercalated pairs (**Figure S15**), is located in the core of the C:C^{+} stack. Thus, starting from tC^{O}6 signals, C14, C1 and C19 could be specifically assigned. Imino-imino cross-peaks between intercalated base pairs also allowed identifying C7:C15^{+} pair. Although due to the overlapping of NOE signals in the non-exchangeable region, C7 and C15 could not be specifically assigned, the observation of a cross-peak C7H41-C19H5 allowed the identification of the amino protons of C7. C20 residue was tentatively assigned based on an unusual H5 chemical shift (5.34 ppm) that may indicate, as previously discussed, the interaction across the major groove with tC^{O}6 aromatic system. Four TH3-GH1 cross-peaks are also observable, suggesting the formation of G:T:G:T tetrads. Guanine imino signals could be specifically assigned on the basis of amino-imino cross-peaks with the stacked cytosines.

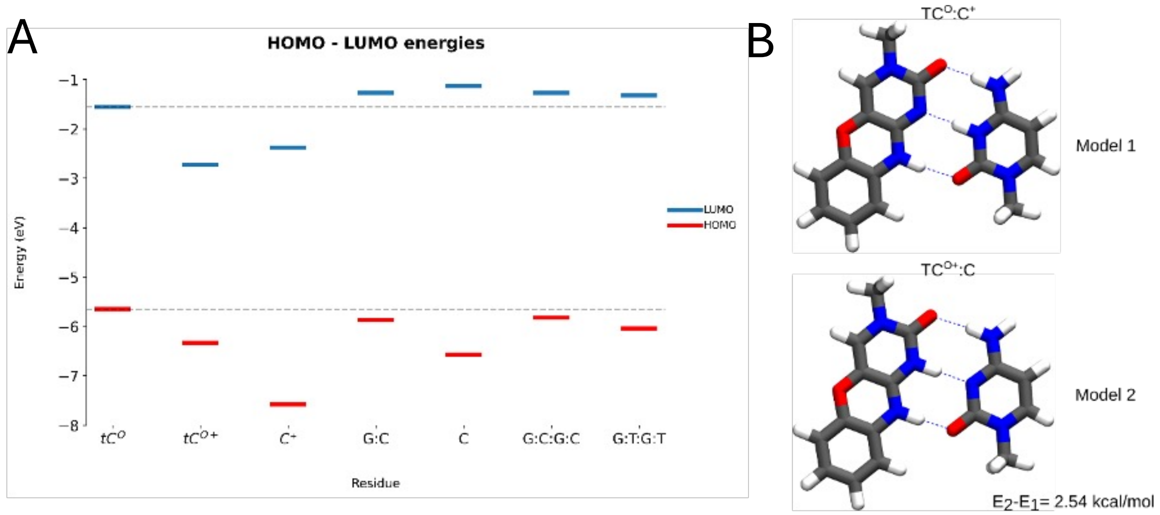

Figure S17. QM calculations. A) Calculated HOMO LUMO energies values (eV) with B3LYP method and 6-31+G(d) basis set in water (PCM model). tC^{O+} and C^{+} represent the protonated states of tC^{O} and C. G:C is the base pair stacked with tC^{O}. G:C:G:C and G:T:G:T are the tetrads from the neutral and acidic states. B) Models used to calculate the stability the different protonation states of the tC^{O}:C base pair. Calculations were performed at the B3LYP/6-311G(d) DFT level of theory.

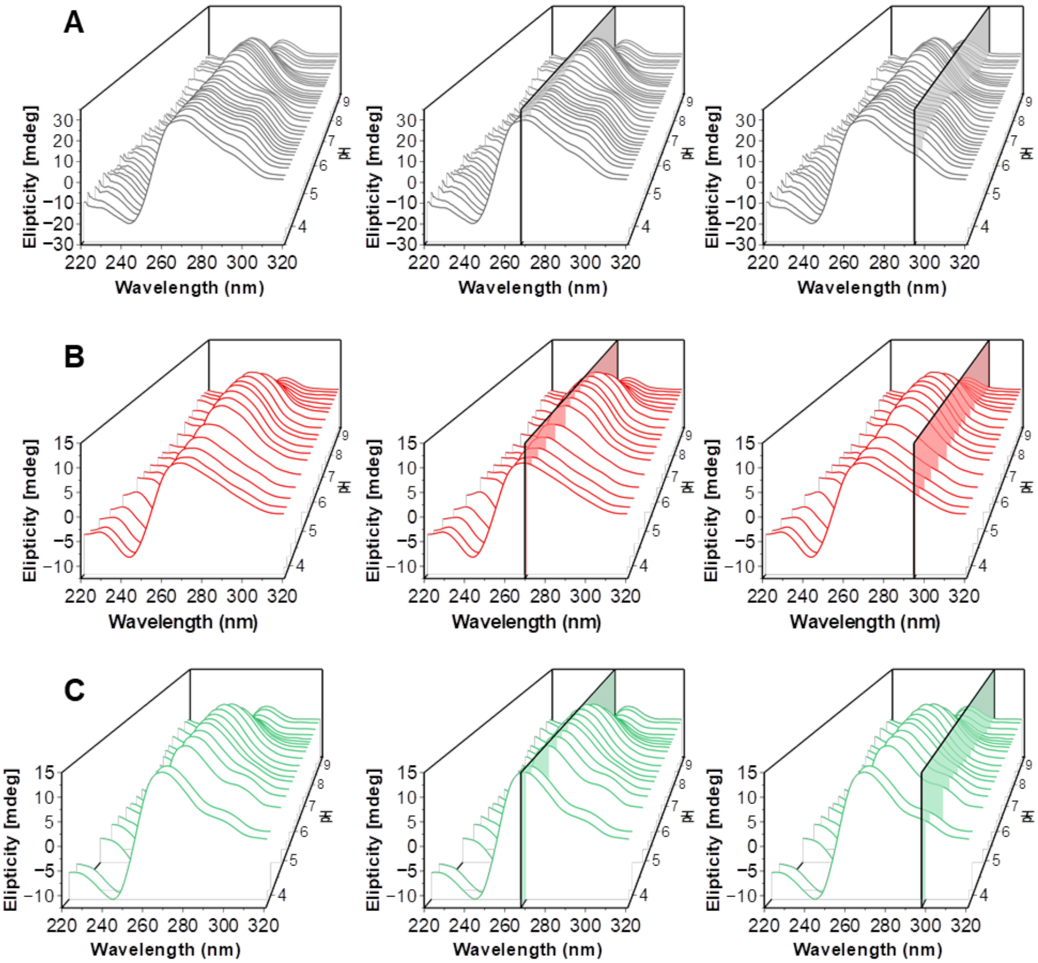

Figure S18. Three-dimensional representation of the CD-monitored pH-titration experiments of **NN4** (A), **NN4_tC^{o}2** (B) and **NN4_tC^{o}6** (C). Ellipticity at different wavelengths is highlighted with a perpendicular plane for each sequence at 270 nm (center) and 295 nm (right). [oligonucleotide] = 2.0 µM, 25 mM sodium phosphate buffer.

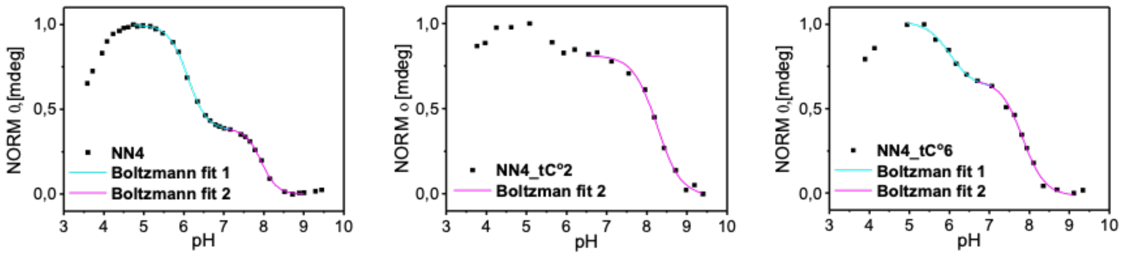

Figure S19.- CD-monitored pH titration curves at 295 nm of **NN4** (left), **NN4_tC^{O}2** (center) and **NN4_tC^{O}6** (right) at 5 °C, 25 mM sodium phosphate buffer, [oligonucleotide] = 2.0 µM. Boltzmann fit represented in magenta for the denaturation of the structures (pH_{T2}) and in cyan for the equilibrium between species (pH_{T1}).

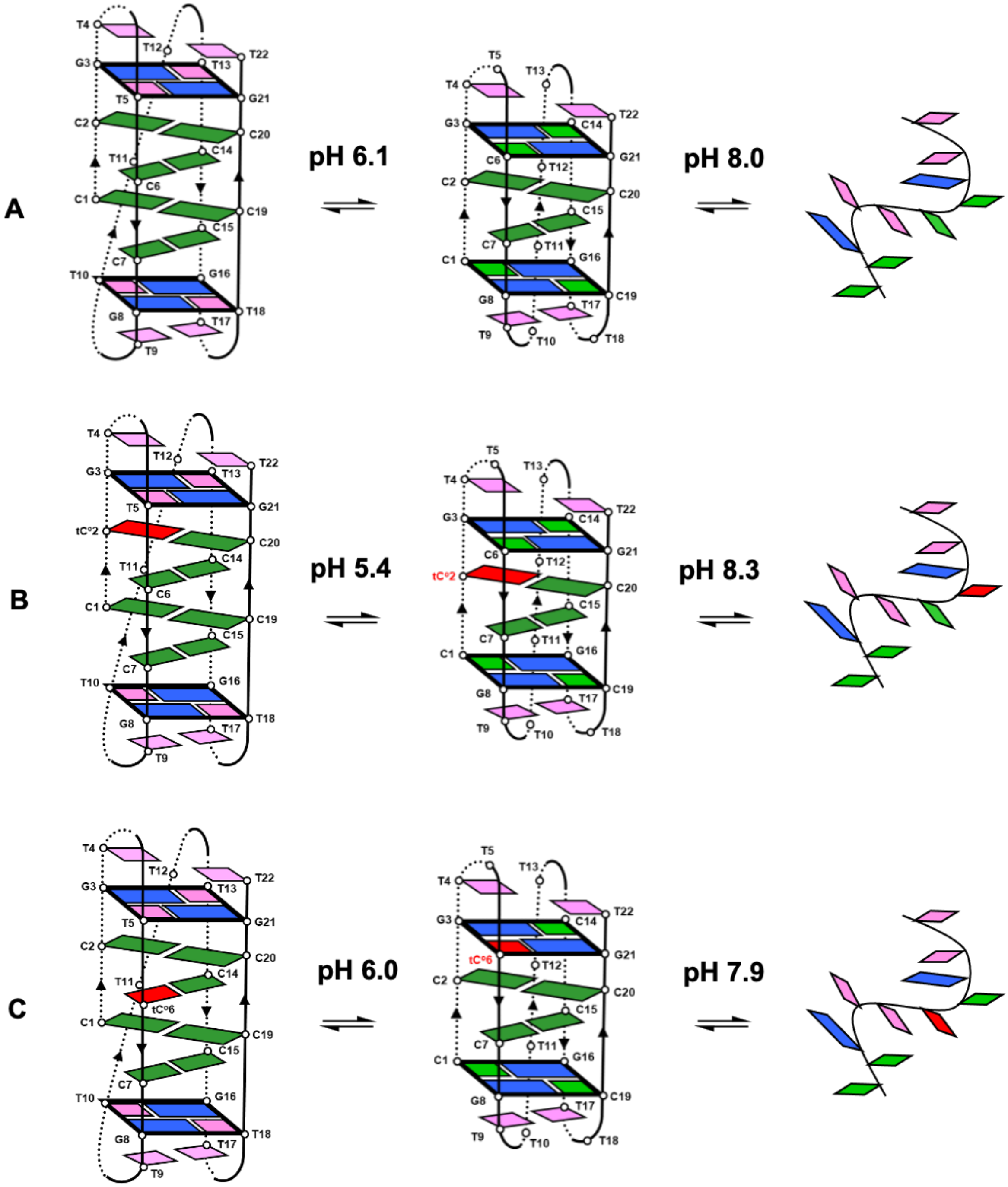

Figure S20.- Summary of the different equilibria observed in **NN4** and its modified analogs with pH_T values obtained by monitoring the CD spectra at 265 and/or 295 nm.

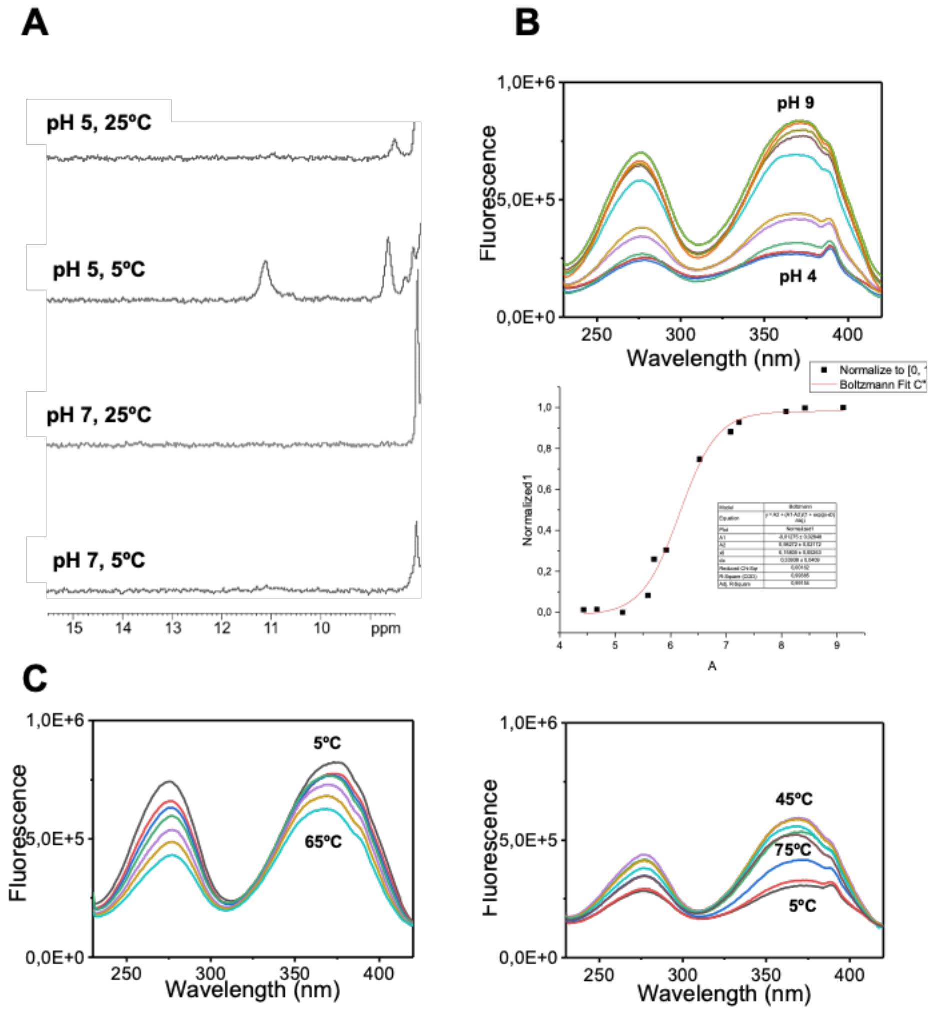

Figure S21. A) ^{1}H-NMR spectra at different pH and temperatures of **ControlNN4_tC^{O}2** (d(GtC^{O}GTTCCTTTTTTGCGTTCCTT)). B) Excitation fluorescence spectra (top) of **ControlNN4_tC^{O}2** at different pH and 5 °C and its sigmoidal fit representation (bottom, pH$_{T}$ = 6.2) at 370 nm. C) Excitation fluorescence spectra of **ControlNN4_tC^{O}2** at different temperatures at pH 7 (left) and pH 5 (right). 10 mM phosphate buffer and [oligonucleotide] = 0.2 uM

A

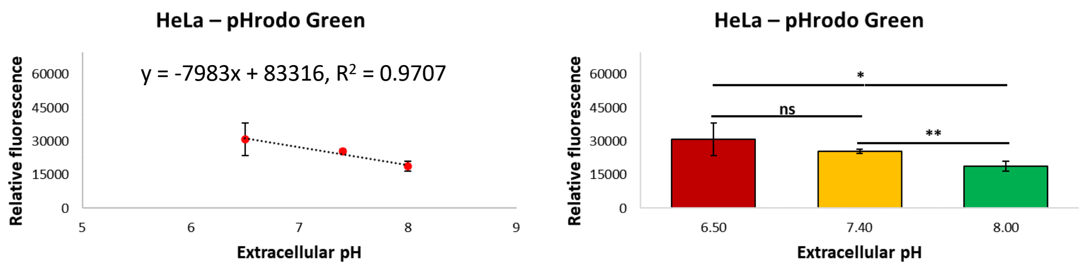

B

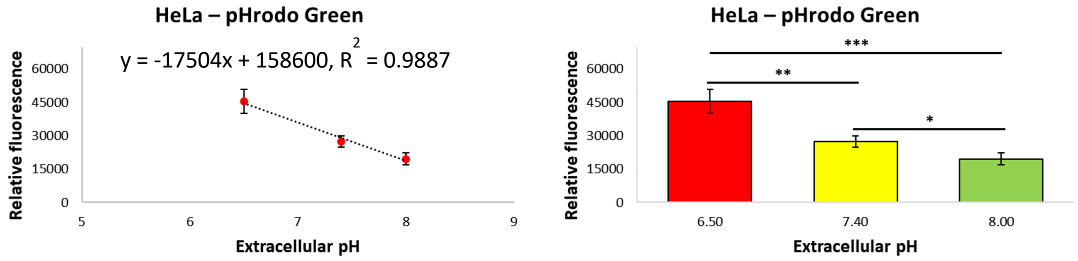

C

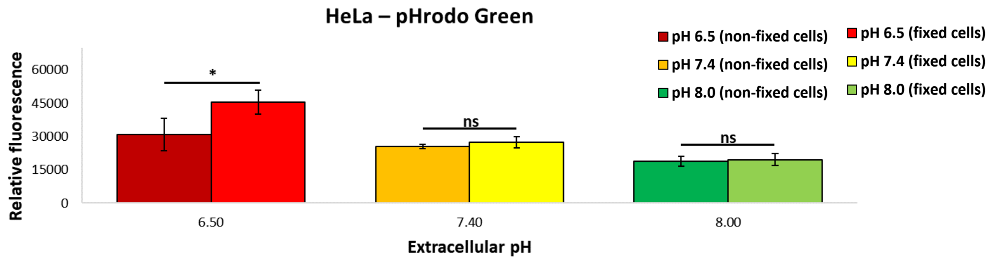

D

	pH (acidic media)	pH (physiological media)	pH (alkaline media)
**FIXED**	6.56	7.41	7.89
**NON-FIXED**	6.57	7.46	7.97

***Figure S22*.- pHrodo™ Green fluorescence calibration**. Non-fixed cells (A), fixed cells (B) and comparison of fixed and non-fixed cells (C). Student's T-tests showed significant differences in the fluorescence intensity of pHrodo™ Green in fixed cells between pH 6.5 and 7.4 (p-value: 0.003616), between pH 6.5 and 8.0 (p-value: 0.000725) and between pH 7.4 and 8.0 (p-value: 0.014826); also substantial differences were observed in the fluorescence intensity of pHrodo™ Green in non-fixed cells between pH 6.5 and 8.0 (p-value: 0.040356) and between pH 7.4 and 8.0 (p-value: 0.004998). The comparison between non-fixed and fixed cells showed statistically significant differences only at pH 6.5 (p-value: 0.040746), possibly due to a decrease in viability for non-fixed cells. Also, there was a good correlation between fluorescence intensity and extracellular pH for both fixed (R^2:0.9707, linear regression) and non-fixed cells (R^2:0.9887, linear regression). Extracellular pH measurement after pHrodo™ Green fluorescence quantification (D). For non-treated cells the measured pH was 7.37.

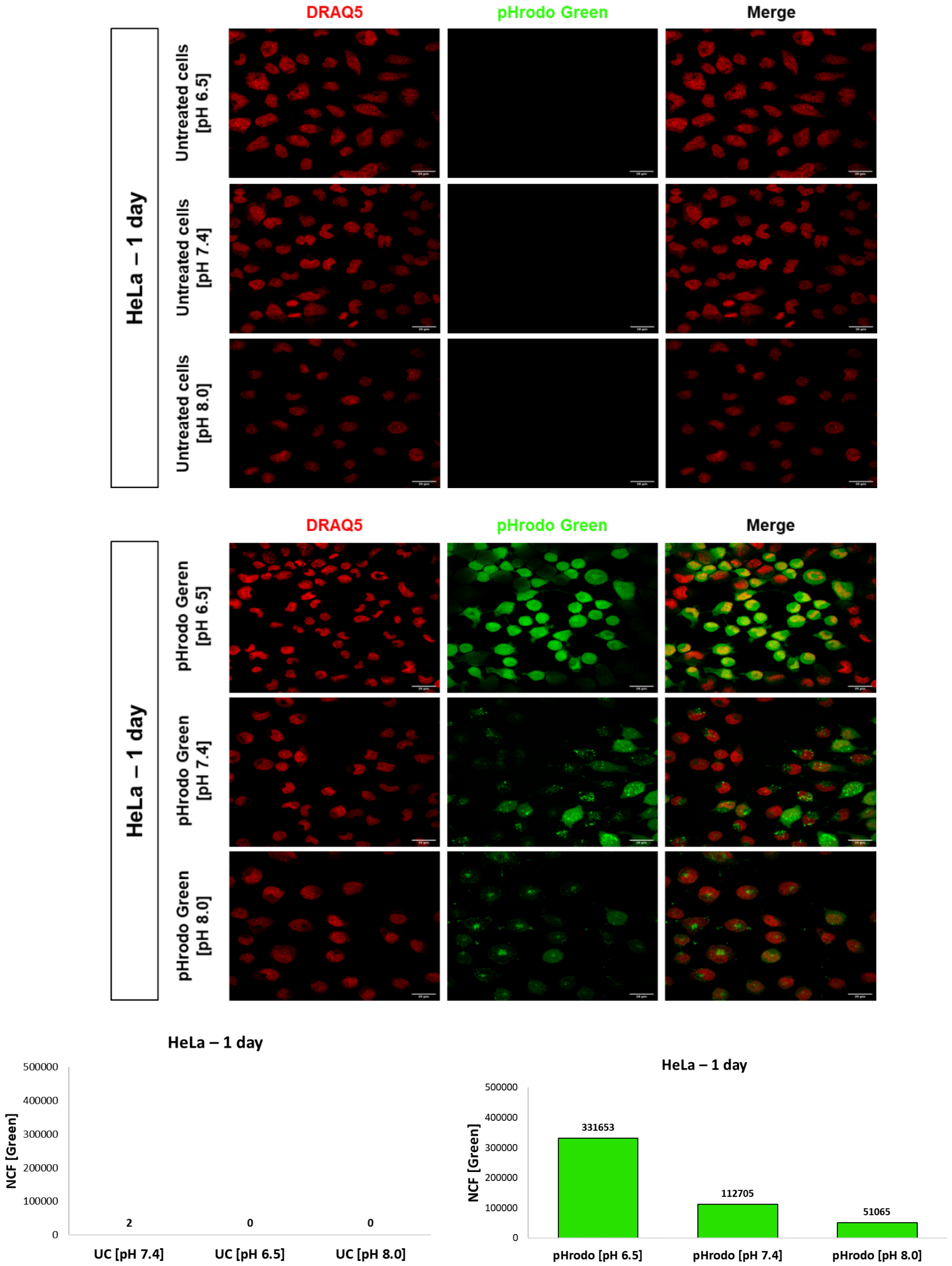

Figure S23. Variation of pHrodoTM Green fluorescence versus pH in fixed cells. Non-treated cells (top), treated cells (middle) and quantification of fluorescence emission in non-treated cells (bottom, left) and treated cells (bottom, right). Fluorescence quantification values have been relativized respect to the average of the signal recorded for untreated cells at each pH.

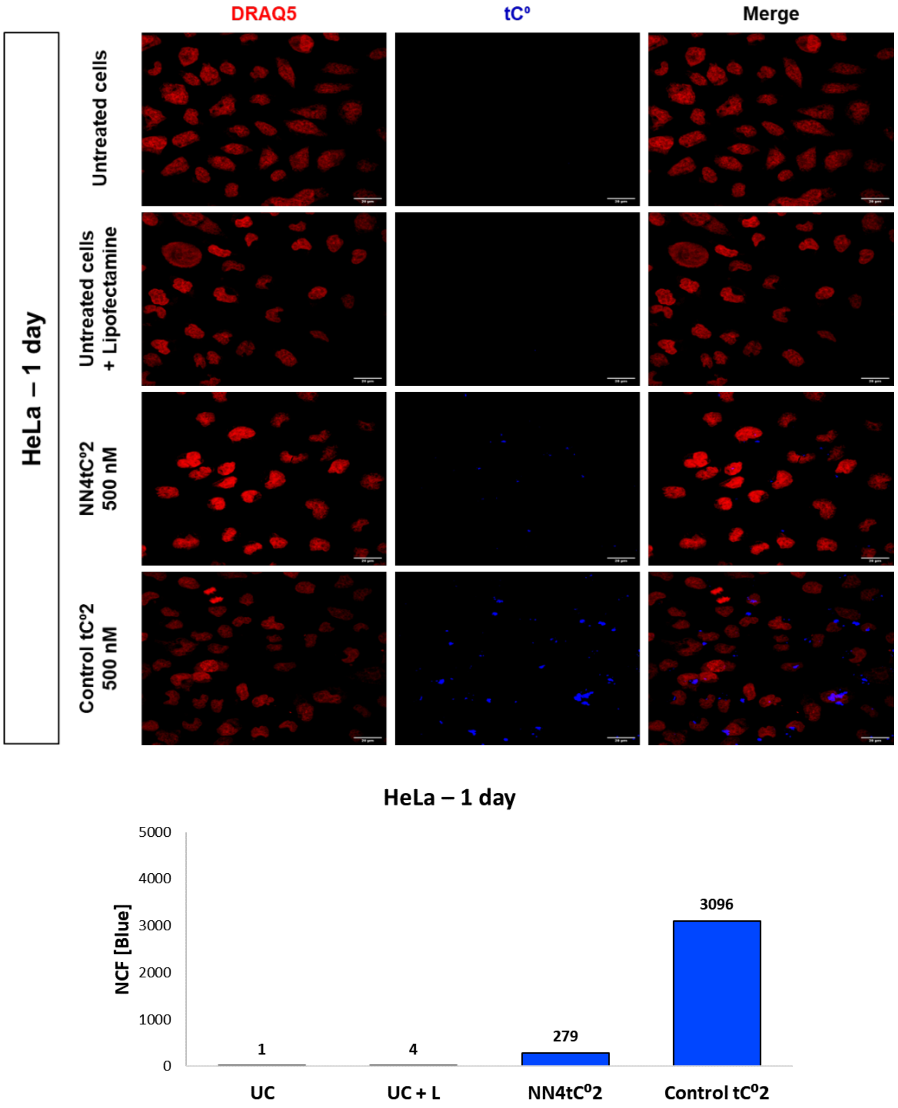

Figure S24. Fluorescence emission of transfected HeLa cells at acidic pH (6.5), compared with non-transfected cells and cells treated with lipofectamine 2000. Fluorescence quantification values have been normalized with respect to the average of the signal recorded for untreated cells.

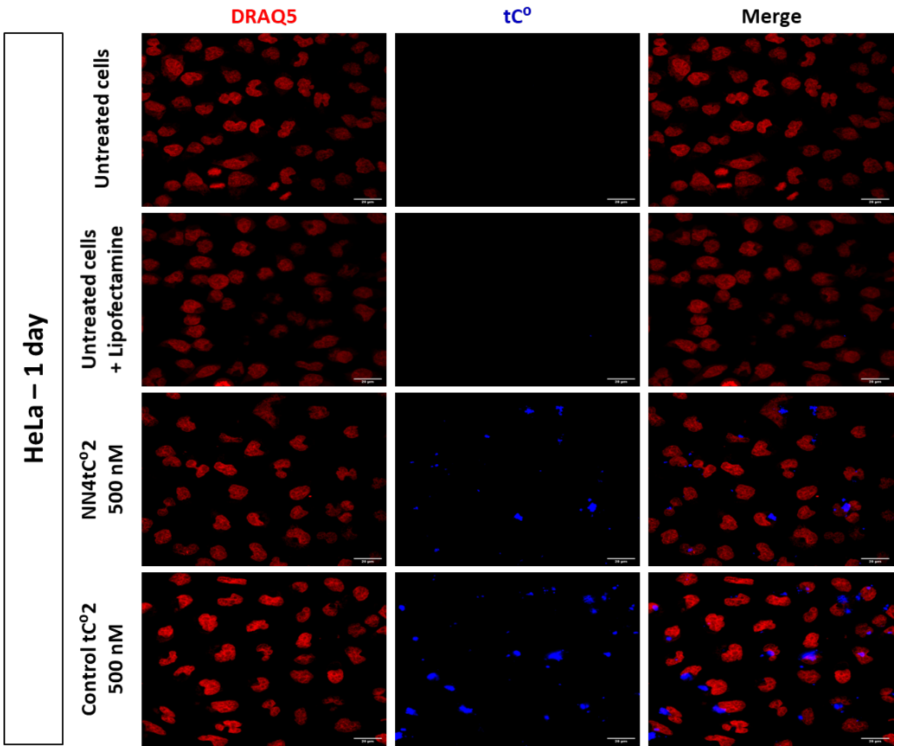

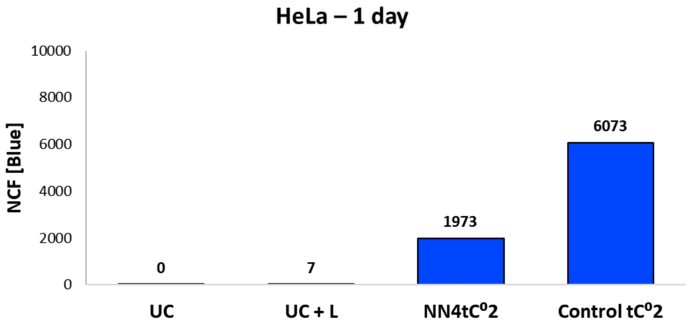

Figure S25. Fluorescence emission of transfected HeLa cells at physiological pH (7.4), compared with non-transfected cells and cells treated with lipofectamine 2000. Fluorescence quantification values have been normalized with respect to the average of the signal recorded for untreated cells.

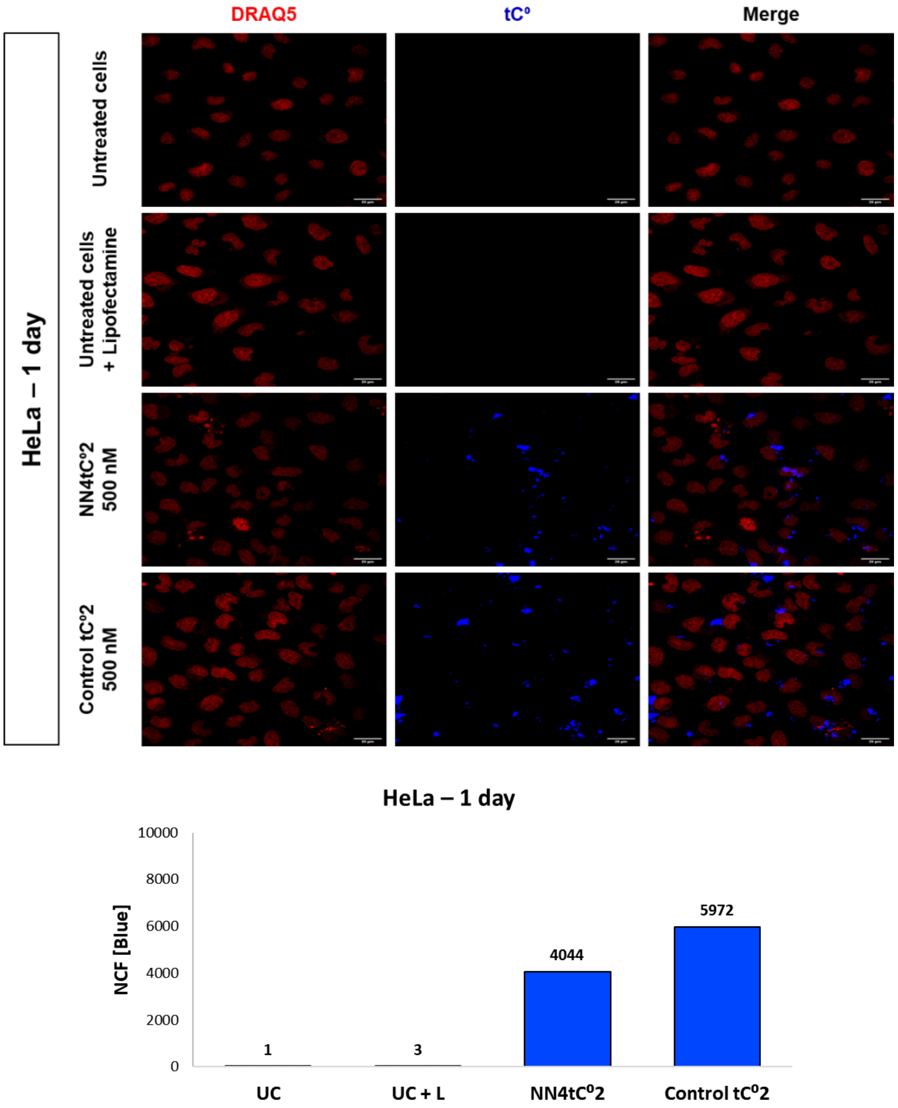

Figure S26. Fluorescence emission of transfected HeLa cells at alkaline pH (8.5), compared with non-transfected cells and cells treated with lipofectamine 2000. Fluorescence quantification values have been normalized with respect to the average of the signal recorded for untreated cells.

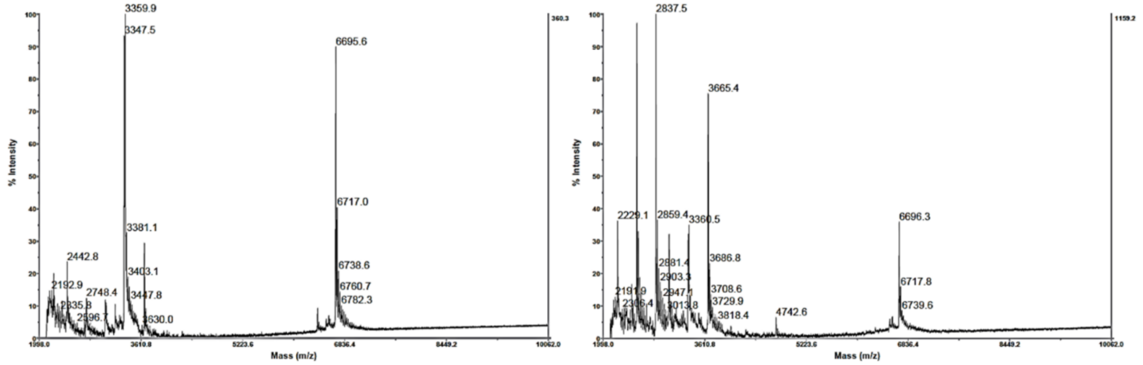

__Figure S27__.**-** MALDI-TOF spectrum of the sequences **NN4_tC^{O}2** (left) and **NN4_tC^{O}6** (right).

Supplementary Tables

Table S1.- *Chemical shifts of* **NN4_tC°2** at pH 7 and T= 5 °C. n.a.: not assigned, n.o.: not observed.

RESIDUE	H1/H3⁺	H42/H22/H10	H41/H21	H6/H8/H4	H5/Me	H1'	H2'	H2"	H3'
C1	-	8.38	7.30	7.85	6.03	6.26	2.51	2.80	4.95
tC°2	15.01	9.82	-	7.18	-	6.04	1.20	1.83	4.80
G3	13.93	8.79	7.05	8.11	-	6.07	2.77	3.06	5.02
T4	n.o.	-	-	7.65	1.78	6.20	2.07	2.32	3.83
T5	n.o.	-	-	7.86	1.94	6.50	2.32	2.56	4.63
C6	-	8.75	7.66	8.02	6.23	6.38	2.46	2.62	4.93
C7	15.57	8.67	8.05	7.64	5.74	6.18	1.24	2.04	4.80.
G8	13.44	8.22	7.19	8.15	-	6.00	3.03	2.69	5.05
T9	n.o.	-	-	7.64	1.74	6.01	2.00	2.30	4.78
T10	n.o.	-	-	7.61	1.80	6.14	2.32	2.58	4.91
T11	n.o.	-	-	7.76	1.93	6.41	2.43	2.57	4.93.
T12	n.o.	-	-	7.78	1.93	6.35	2.38	2.62	4.95.
T13	n.o.	-	-	7.81	2.00	6.45	2.42	2.53	5.06
C14	-	8.62	6.37	7.75	5.54	6.33	2.27	2.67	4.93
C15	15.57	10.83	n.o.	7.52	5.31	6.02	0.88	2.41	4.44
G16	12.82	n.o.	n.o.	8.21	-	6.11	2.97	2.65	5.04
T17	n.o.	-	-	7.53	1.74	6.11	2.00	2.39	4.77
T18	n.o.	-	-	7.82	1.93	6.43	2.11	2.56	4.70
C19	-	8.74	7.22	7.85	6.03	6.43	2.33	2.61	4.88
C20	15.01	11.34	8.86	7.53	6.02	6.23	0.96	2.54	4.35
G21	13.37	8.22	7.19	8.27	-	6.12	2.98	2.64	5.03
T22	n.o.	-	-	7.57	1.74	5.76	1.90	2.10	4.36

RESIDUE	H6	H7	H8	H9
tC°2	6.07	6.39	6.66	5.92

Table S2.- *Chemical shifts of non-exchangeable protons of* **NN4_tC^{o}2** at pH 7 and T= 20 ^{o}C. n.a.: not assigned, n.o.: not observed

RESIDUE	H6/H8/H4	H5/Me	H1’	H2’	H2”	H3’	H4’	H5’/H5”
C1	7.86	6.05	6.25	2.52	2.79	4.94	n.a.	3.88/3.92
tC^{o}2	7.21	-	6.02	1.22	1.83	4.79	4.50	4.09/4.17
G3	8.08	-	6.05	2.76	3.04	5.01	4.57	3.89/4.08
T4	7.64	1.77	6.19	2.06	2.33	4.78	n.a.	n.a.
T5	7.85	1.93	6.48	2.31	2.56	4.60	n.a.	n.a.
C6	8.01	6.23	6.36	2.45	2.61	4.92	4.39	3.81/3.98
C7	7.66	5.74	6.16	1.25	2.00	4.79	4.50	4.07/4.15
G8	8.10	-	5.99	3.01	2.69	5.04	4.52	3.98/4.08
T9	7.62	1.73	6.01	1.99	2.31	4.79	n.a.	n.a.
T10	7.62	1.62	6.18	2.34	2.55	4.91	n.a.	n.a.
T11	7.73	1.93	6.38	2.41	2.56	4.92	n.a.	n.a.
T12	7.75	1.93	6.34	2.37	2.61	4.93	n.a.	n.a.
T13	7.79	1.99	6.42	2.41	2.52	5.05	n.a.	n.a.
C14	7.72	5.51	6.34	2.25	2.67	4.93	4.32	n.a.
C15	7.46	5.18	6.00	0.86	2.41	4.40	4.07	3.94/n.a.
G16	8.18	-	6.10	2.95	2.63	5.02	4.52	4.03/4.09
T17	7.52	1.72	6.01	1.98	2.40	4.77	4.52	3.76/3.88
T18	7.80	1.92	6.41	2.11	2.54	4.69	n.a.	n.a.
C19	7.82	6.01	6.44	2.30	2.60	4.87	4.28	n.a.
C20	7.52	5.99	6.20	0.98	2.54	4.31	n.a.	n.a.
G21	8.25	-	6.11	2.96	2.63	5.01	4.45	4.05
T22	7.56	1.73	5.76	1.90	2.17	4.34		3.65/3.82

RESIDUE	H6	H7	H8	H9
tC^{o}2	6.09	6.31	6.66	5.89

Table S3.- Experimental constraints and calculation statistics of **NN4_tC°2** at pH 7.

Experimental distance constraints		
Total number	115	
intra-residue	42	
sequential	38	
range > 1	35	
**RMSD (Å)**		
all well-defined* bases	0.6 ± 0.2	
all well-defined* heavy atoms	0.9 ± 0.2	
backbone	1.6 ± 0.4	
all heavy atoms	2.2 ± 0.4	
**Residual violations**	Average	Range
Sum of violation (Å)	2.88	2.29 ... 3.23
Max. violation (Å)	0.42	0.33 ... 0.49
NOE energy# (kcal/mol)	23.1	20.4 ... 25.7
Total energy (kcal/mol)	- 916	- 996 ... - 875
* All except thymines 5,10,11,12,13,18		
# K_{NOE} = 20 kcal/(mol·Å^2)		

Table S4.- Average dihedral angles and order parameters of the structure of **NN4_tC°2**. Average values correspond to the geometrical mean value and order parameters are use as indicators of angle definition: S=1, perfectly defined and S=0, random distribution.

Nt	α		β		γ		δ		ε		ζ		χ	
	Avg.	OP	Avg.	OP	Avg.	OP	Avg.	OP	Avg.	OP	Avg.	OP	Avg.	OP
C1	-	-	-	-	-57	0.6	121	1.0	-94	1.0	-	-	-93	1.0
tC°2	-76	1.0	151	1.0	42	1.0	142	1.0	-180	1.0	164	0.9	-116	1.0
G3	-78	1.0	-161	1.0	58	1.0	148	0.9	-161	1.0	-98	1.0	-85	1.0
T4	-71	1.0	179	1.0	57	1.0	150	1.0	-129	1.0	-179	0.9	-114	1.0
T5	89	0.9	-168	0.8	74	0.7	153	1.0	-111	0.8	60	1.0	-132	1.0
C6	-62	1.0	-176	1.0	59	1.0	143	1.0	-114	0.9	-92	0.7	-115	1.0
C7	-76	1.0	163	1.0	53	1.0	148	1.0	-171	1.0	176	1.0	-131	1.0
G8	-71	1.0	-164	1.0	54	1.0	145	1.0	-160	1.0	-87	1.0	-70	1.0
T9	-68	1.0	171	1.0	53	1.0	147	1.0	-143	0.9	153	1.0	-119	1.0
T10	-176	1.0	162	0.9	54	1.0	148	1.0	-161	1.0	-168	0.7	-127	1.0
T11	-74	1.0	171	1.0	57	1.0	141	1.0	-169	0.9	-79	1.0	-107	1.0
T12	-91	0.7	169	1.0	68	0.9	145	1.0	-146	0.8	-95	0.8	-126	1.0
T13	-101	0.8	176	0.9	55	1.0	149	1.0	-137	0.9	-164	0.5	-134	1.0
C14	-171	1.0	171	1.0	54	1.0	145	1.0	-153	1.0	-84	0.9	-120	1.0
C15	-32	0.6	-104	0.6	-54	0.9	151	1.0	-167	1.0	-95	0.9	-98	1.0
G16	-73	1.0	167	1.0	59	1.0	141	0.9	-130	0.8	-85	1.0	-91	1.0
T17	-63	1.0	151	1.0	49	1.0	134	1.0	-109	1.0	159	0.7	-163	1.0
T18	79	1.0	155	1.0	60	1.0	151	1.0	-161	0.9	60	1.0	-125	1.0
C19	-152	0.9	176	0.9	64	1.0	101	1.0	179	1.0	71	0.7	-117	1.0
C20	-56	1.0	-179	1.0	69	1.0	130	1.0	-153	1.0	-97	0.9	-126	1.0
G21	-68	1.0	173	1.0	62	1.0	149	1.0	-151	1.0	-78	1.0	-86	1.0
T22	-64	1.0	154	1.0	52	1.0	148	1.0	-	-	140	1.0	-131	1.0

Table S5.- Pseudorotation angle and amplitude values that allow the description of the puckering of the ribose ring in the calculated structure of **NN4_tC^{O}2**.

Nt	Pseudorot.		Sugar Conformation
	Phase	Ampl.	
C1	38.7	119.4	C1'-exo
tC^{o}2	39.7	146.5	C2'-endo
G3	34.9	189.1	C3'-exo
T4	38.3	157.8	C2'-endo
T5	35.1	165.5	C2'-endo
C6	31.3	161.3	C2'-endo
C7	39.7	160.1	C2'-endo
G8	34.6	198.7	C3'-exo
T9	37.7	169.0	C2'-endo
T10	36.4	162.8	C2'-endo
T11	32.2	146.0	C2'-endo
T12	40.6	155.1	C2'-endo
T13	38.2	164.8	C2'-endo
C14	35.5	161.3	C2'-endo
C15	40.0	160.3	C2'-endo
G16	38.0	152.4	C2'-endo
T17	48.2	136.7	C1'-exo
T18	40.5	164.3	C2'-endo
C19	29.2	0.4	C3'-endo
C20	27.0	137.1	C1'-exo
G21	29.8	192.3	C3'-exo
T22	39.6	163.9	C2'-endo

***Table S6*.-** Molar extinction coefficients of **NN4** sequences.

SEQUENCE	ε (ml·µmol-1·cm-1)
**NN4**	183.7
**NN4_tC^{O}2**	186.1
**NN4_tC^{O}6**	186.1

***Table S7*.-** Calculated HOMO-LUMO energies values (eV) in water.

	**tC^{O}**	**tC^{O+}**	**C^{+}**	**G:C**	**C**	**G:C:G:C**	**G:T:G:T**
LUMO	-1.55	-2.72	-2.37	-1.27	-1.13	-1.27	-1.32
HOMO	-5.65	-6.33	-7.57	-5.87	-6.57	-5.83	-6.04
